# Supplementary material for: Peroxiredoxin 5 regulates osteogenic differentiation through interaction with hnRNPK during bone regeneration
Source: eLife. 2023 Feb 3;12:e80122. doi: 10.7554/eLife.80122 (PMC9897727; doi:10.7554/eLife.80122)
Supplement: Figure 1—source data 1. [file elife-80122-fig1-data1.docx]

**Figure 1B – source data**

**B**

**0**

**4**

**-**

**+**

**7**

**-**

**+**

**14**

**-**

**+**

**21**

**-**

**+**

**(days)**

**BMP2**

**-**


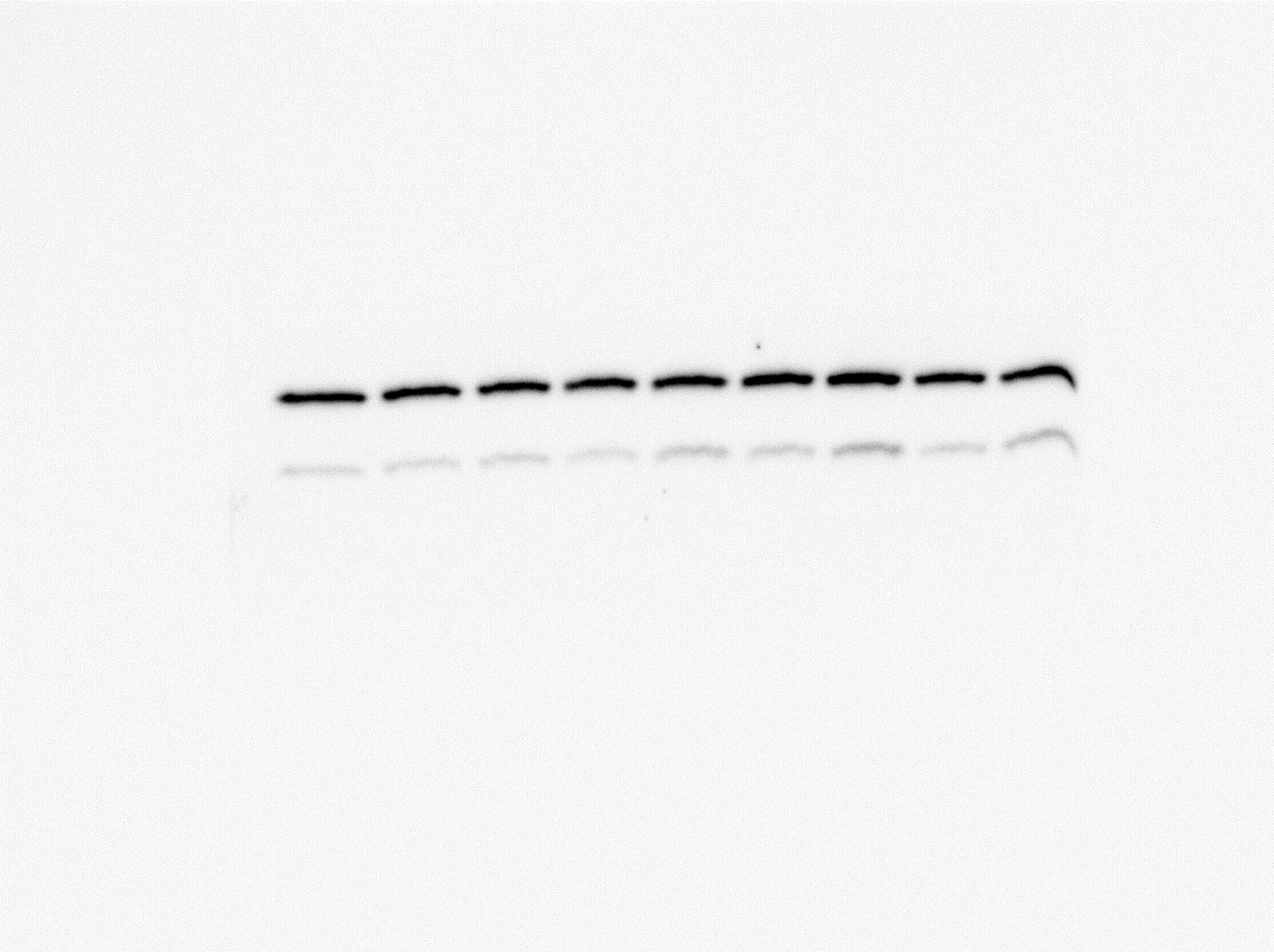


**Prdx1**


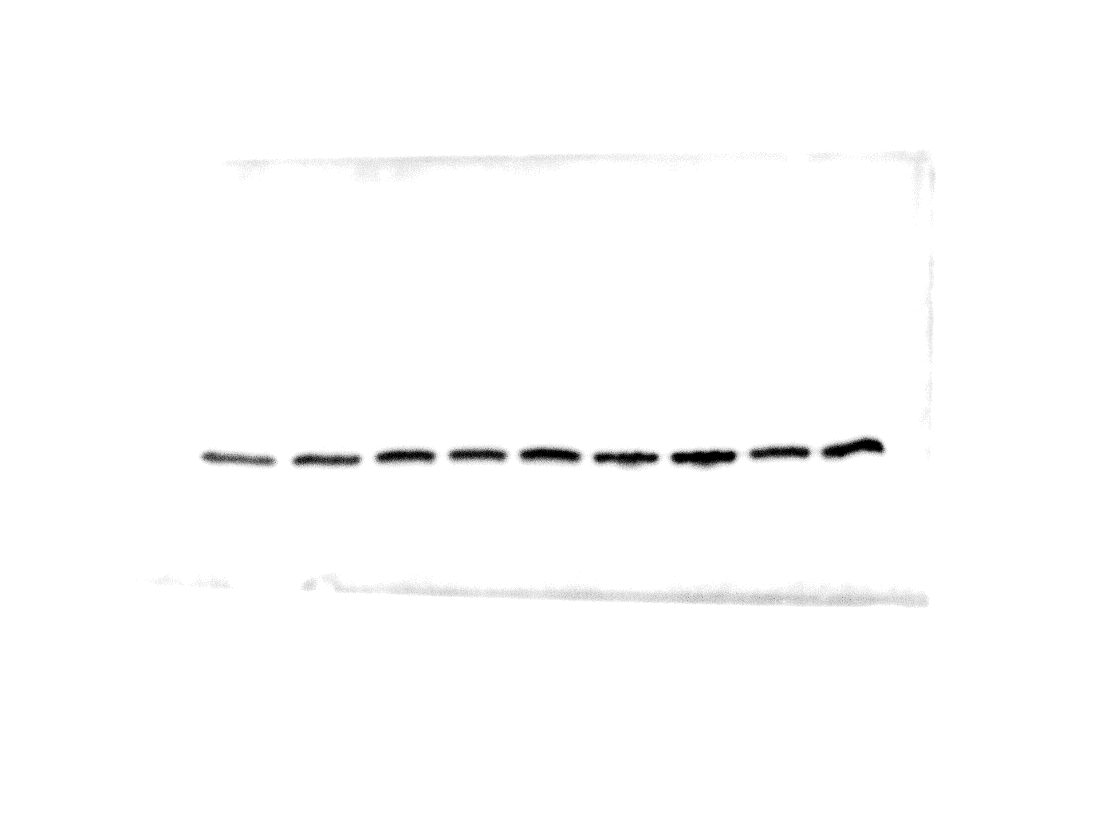


**Prdx2**


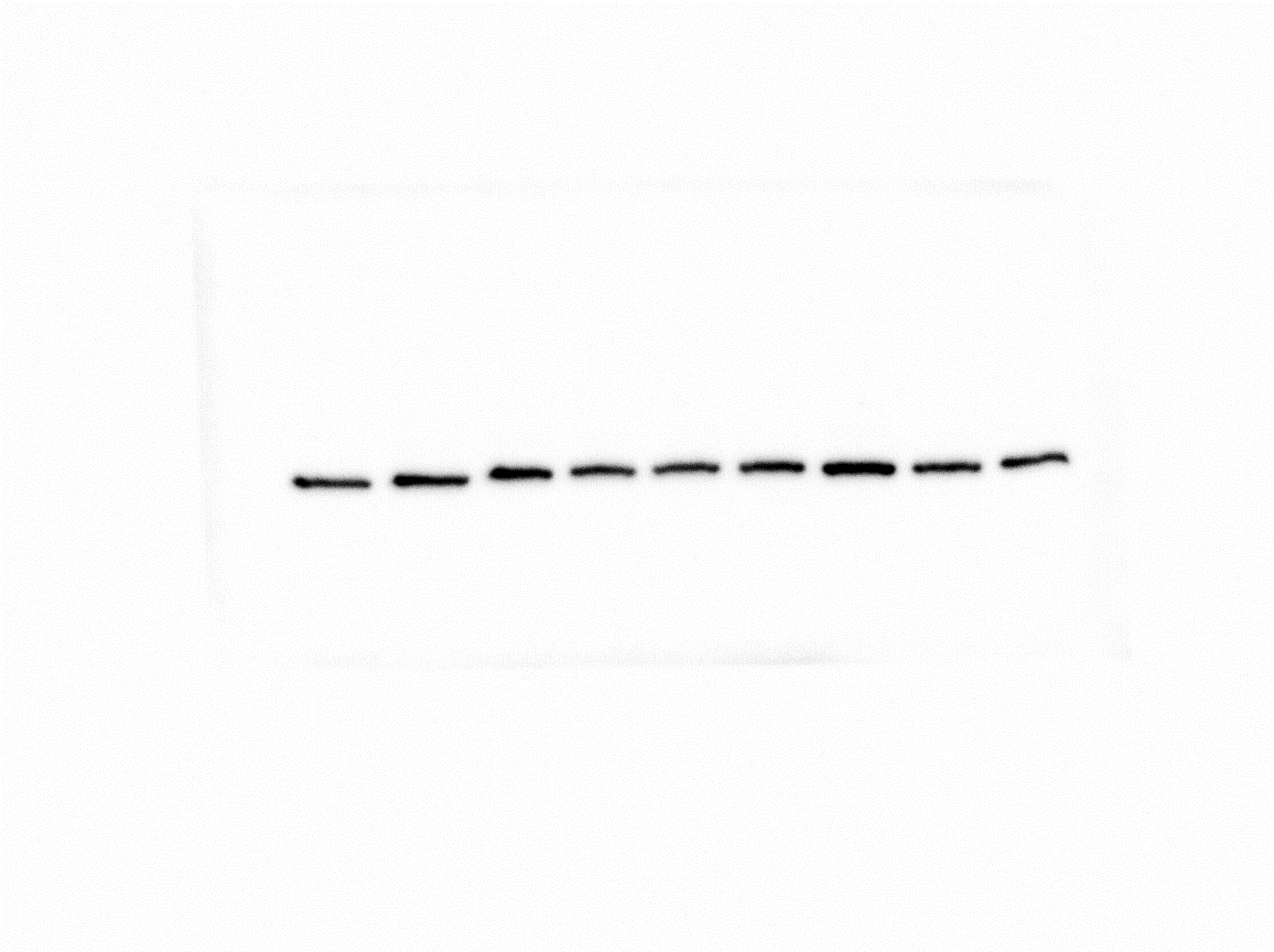


**Prdx3**


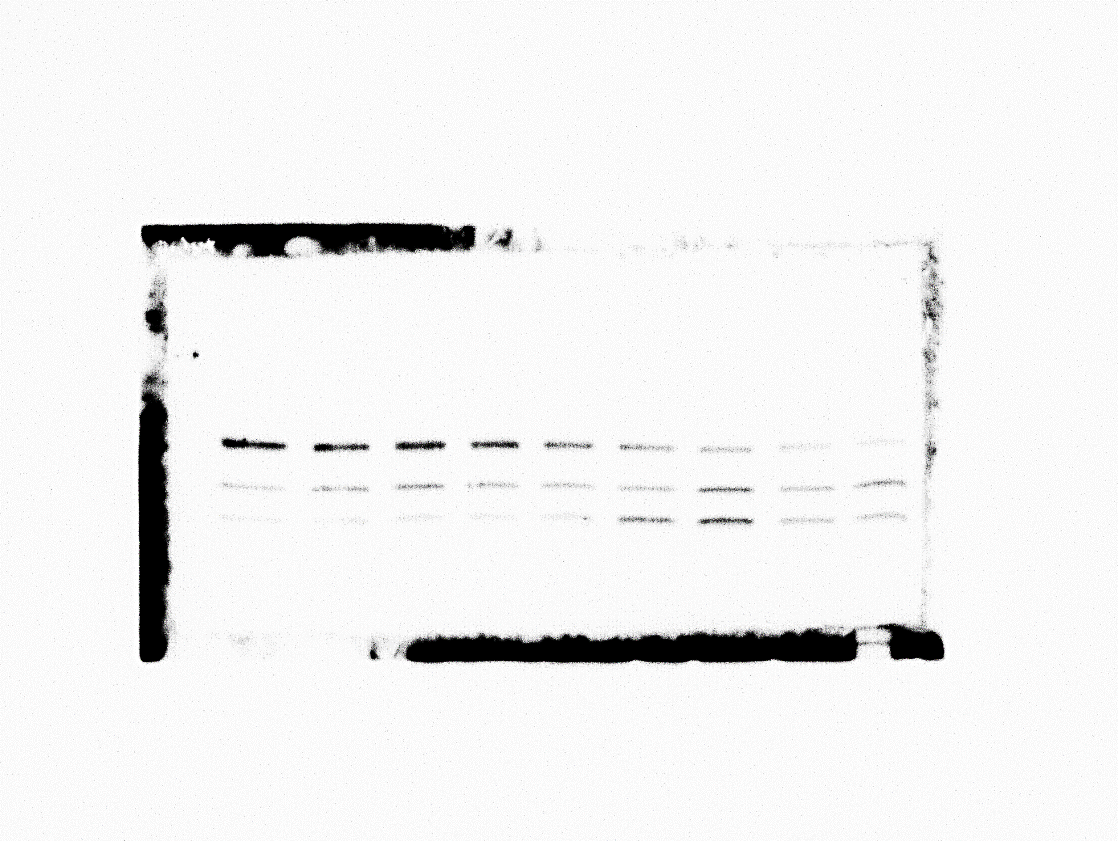


**Prdx4**


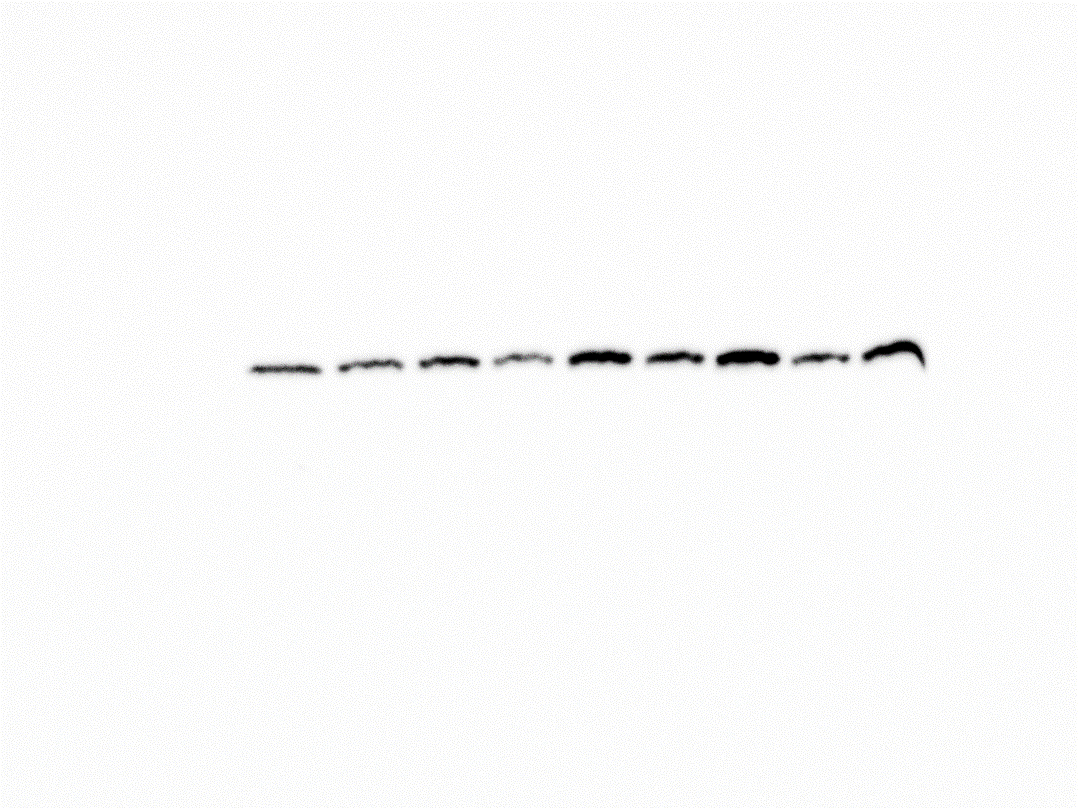


**Prdx5**


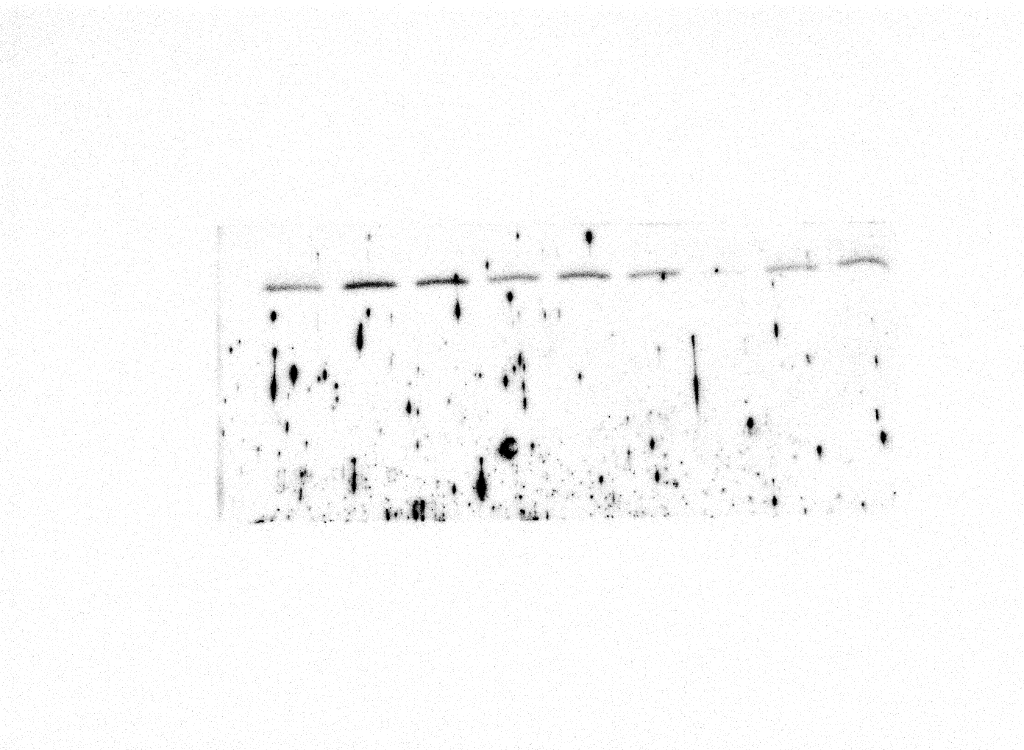


**Prdx6**


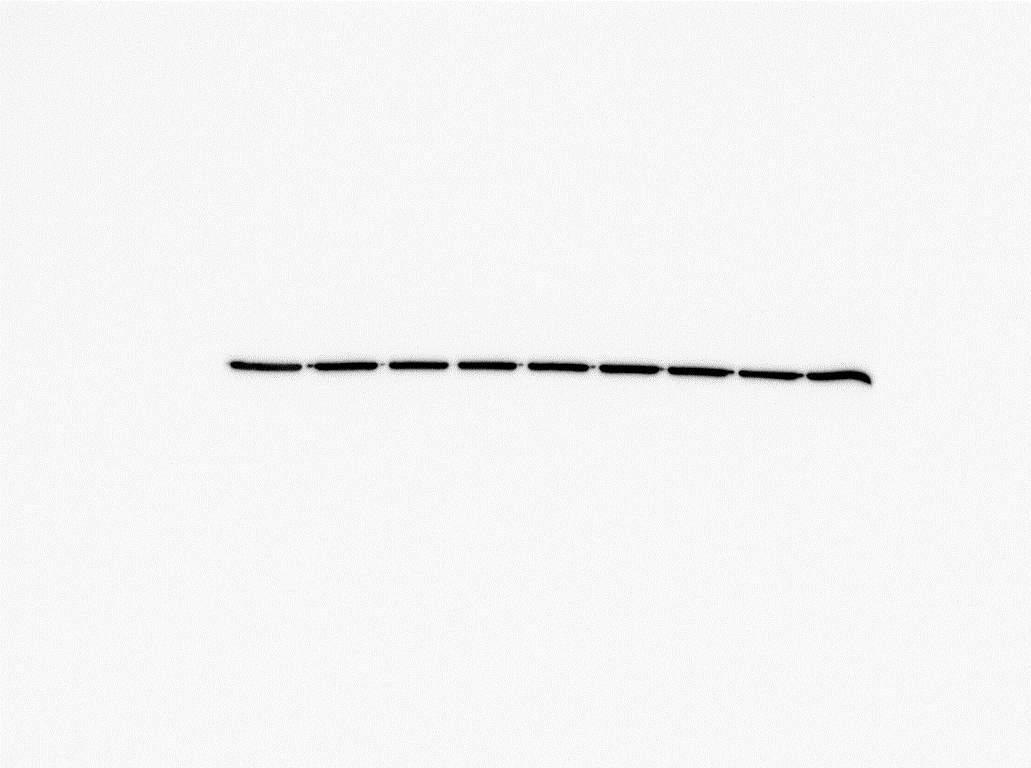


**β-actin**

**Figure 1D – source data**


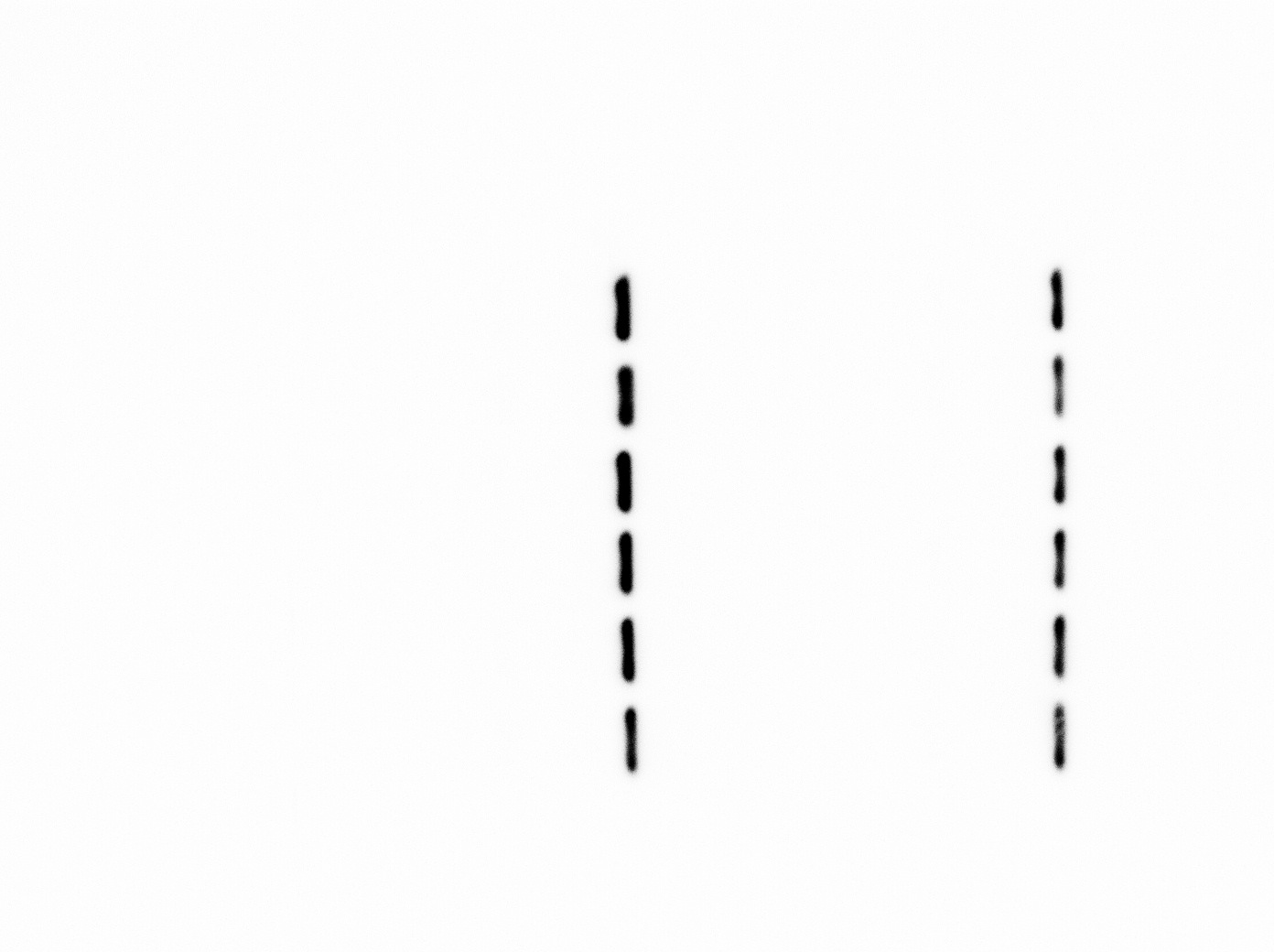

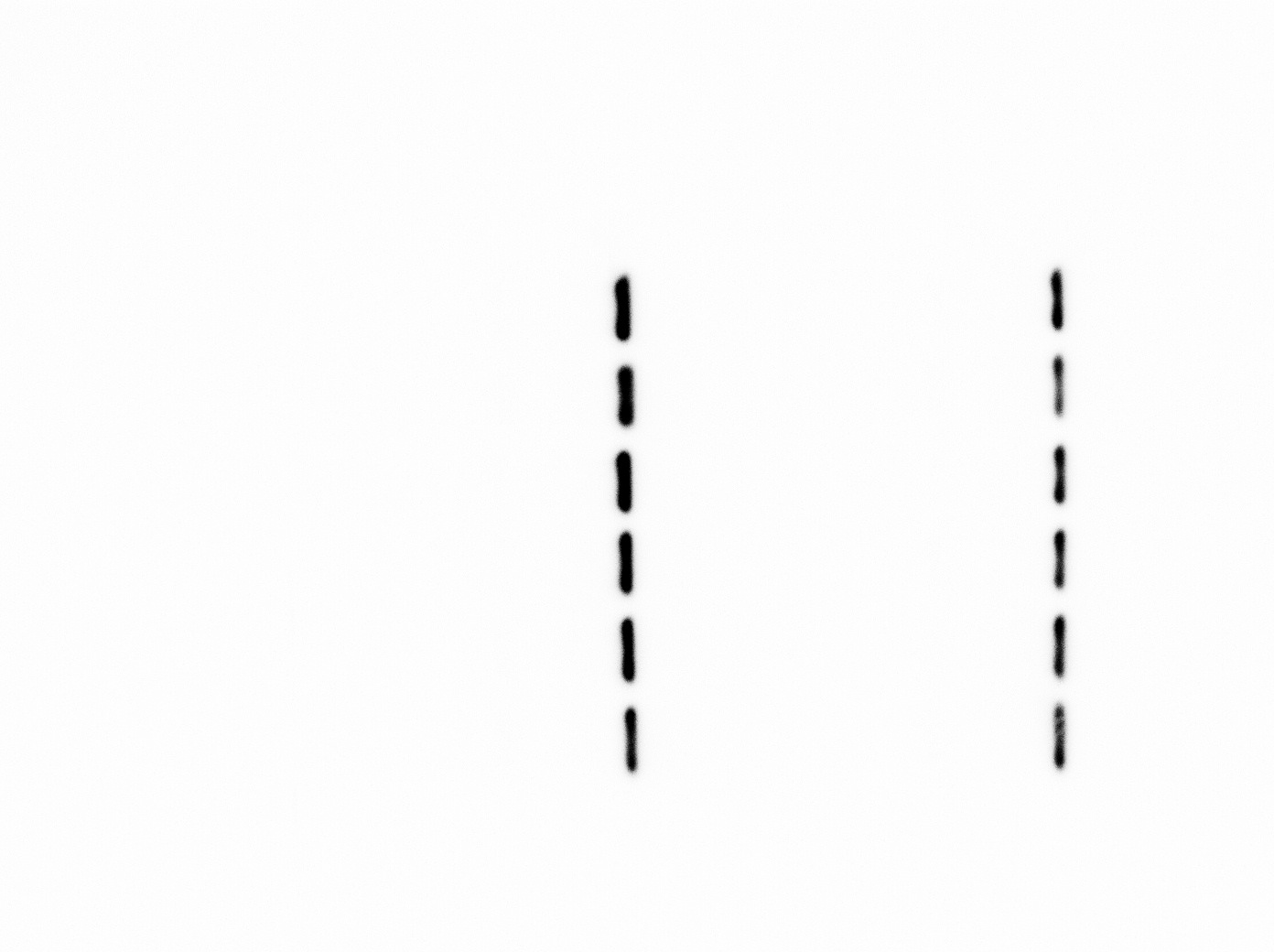


**D**

**0**

**+ RANKL**

**1**

**2**

**3**

**4**

**5**

**(days)**

**0**

**- RANKL**

**1**

**2**

**3**

**4**

**5**


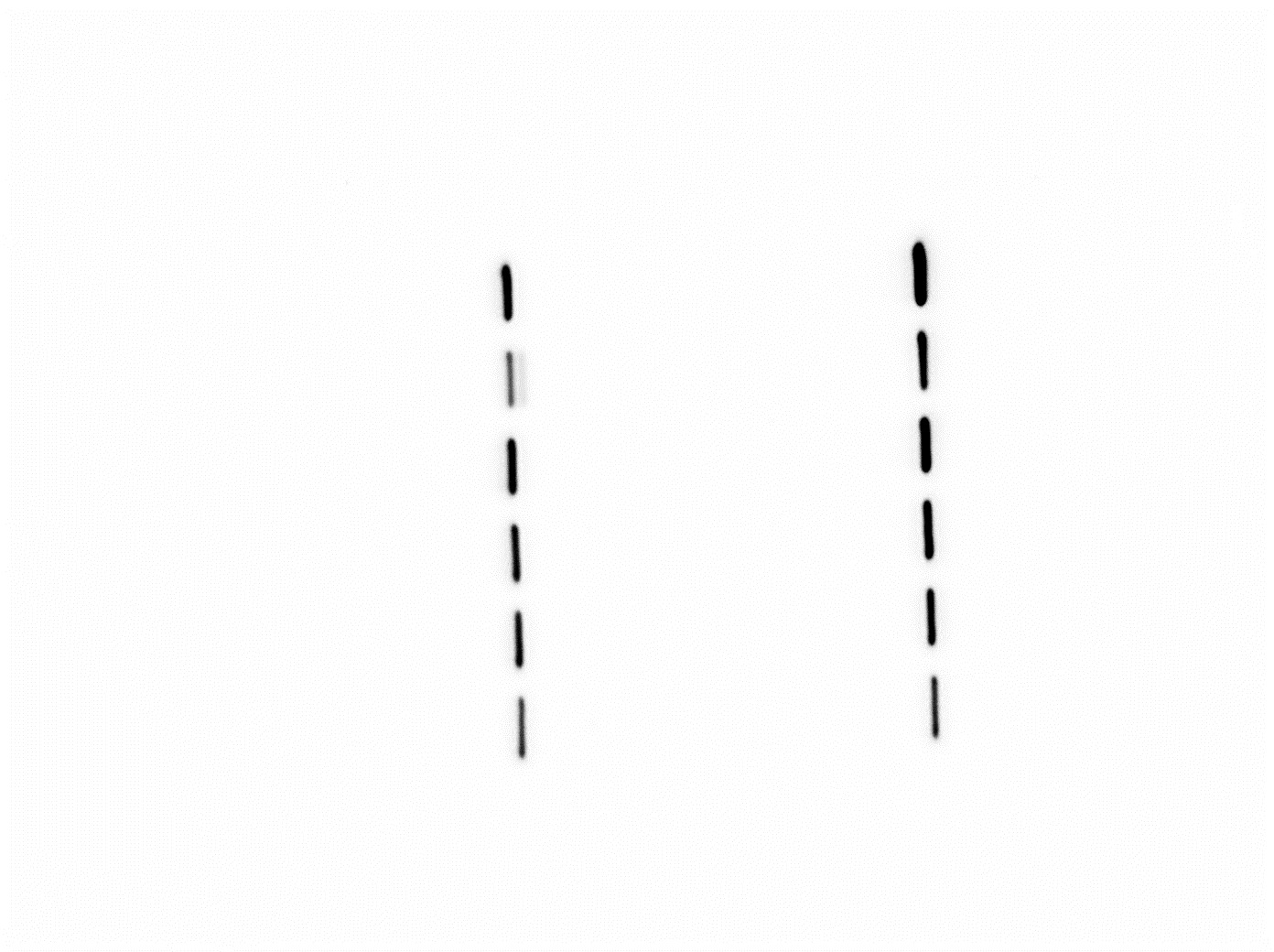

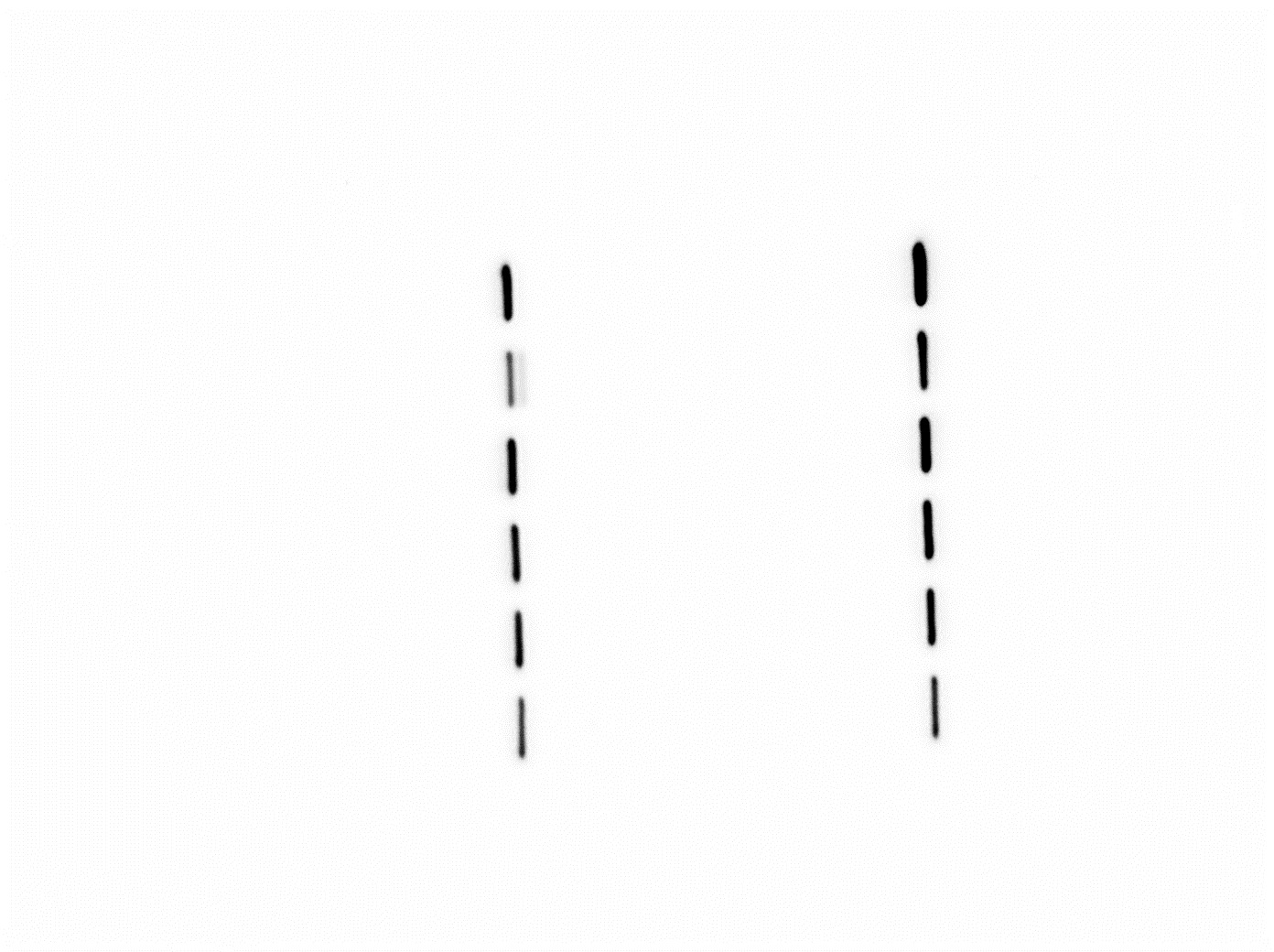


**Prdx1**


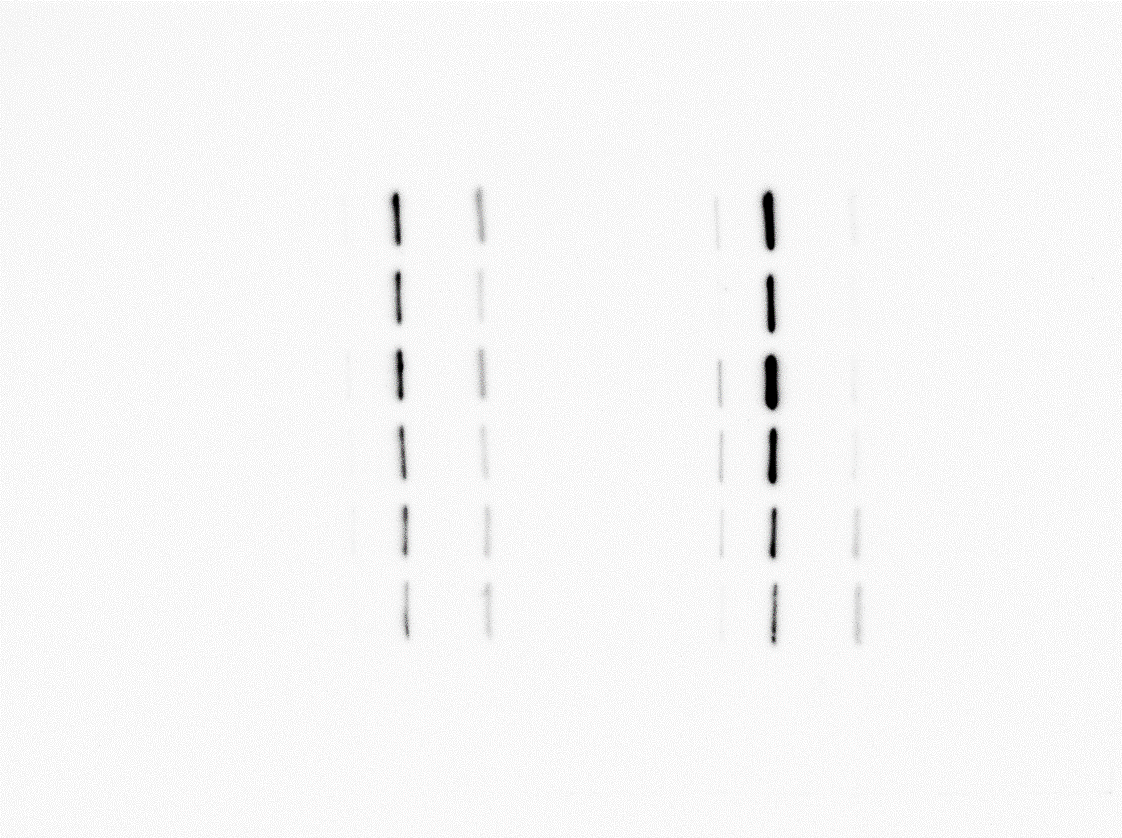

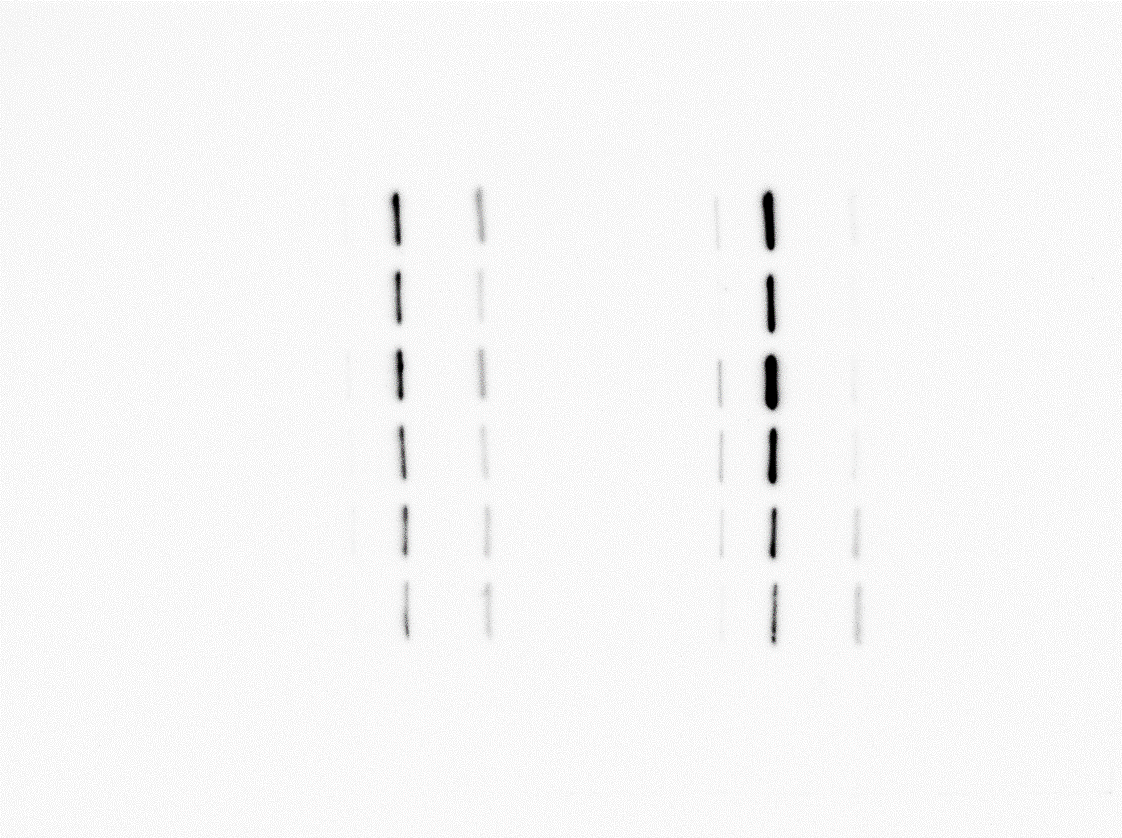


**Prdx2**


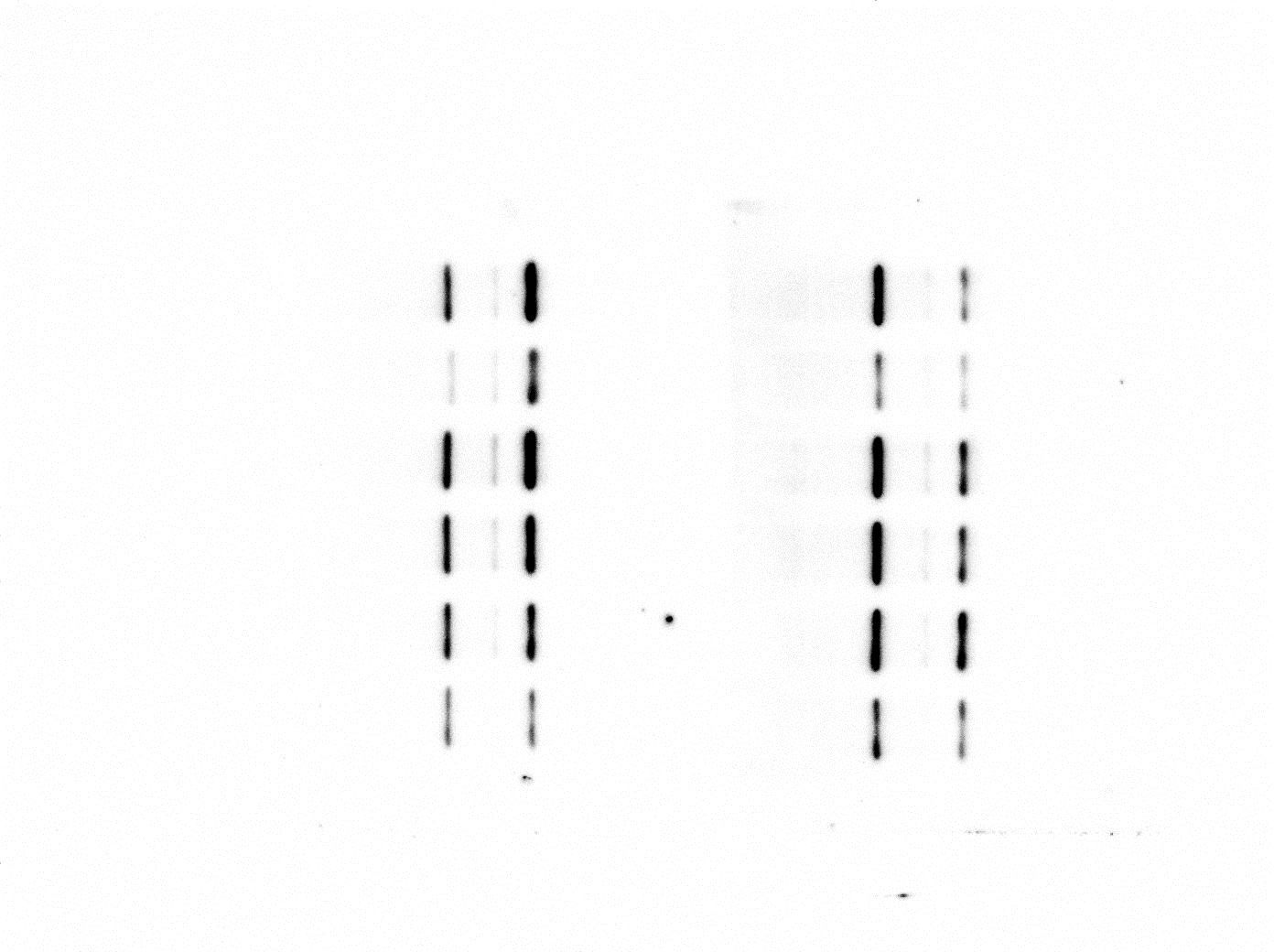

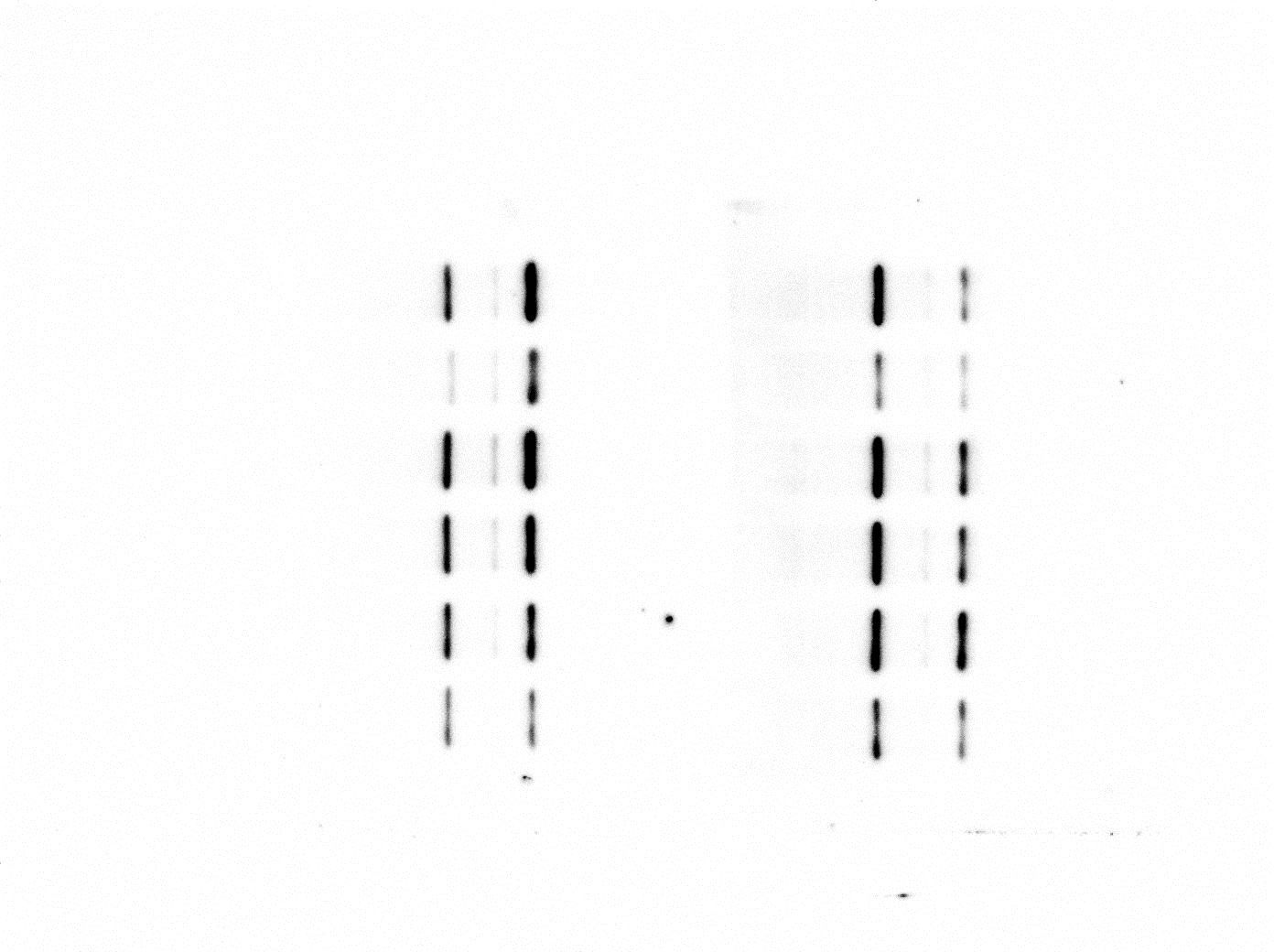


**Prdx3**


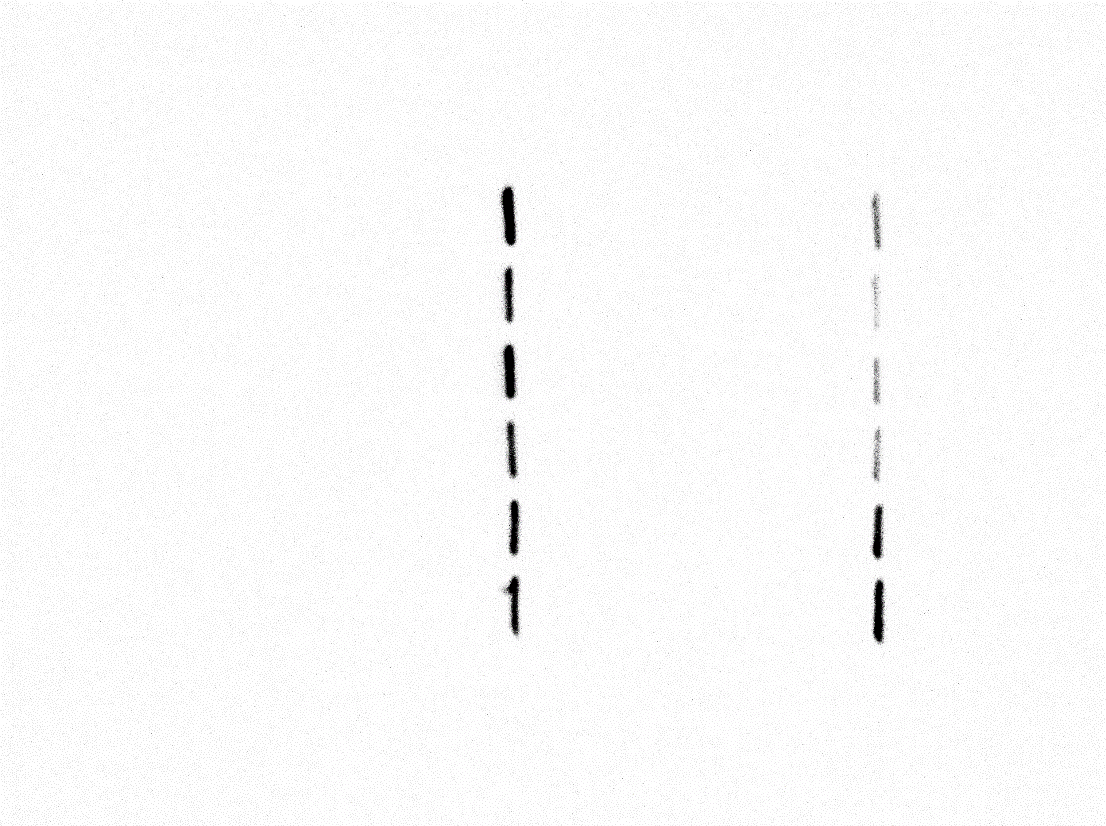

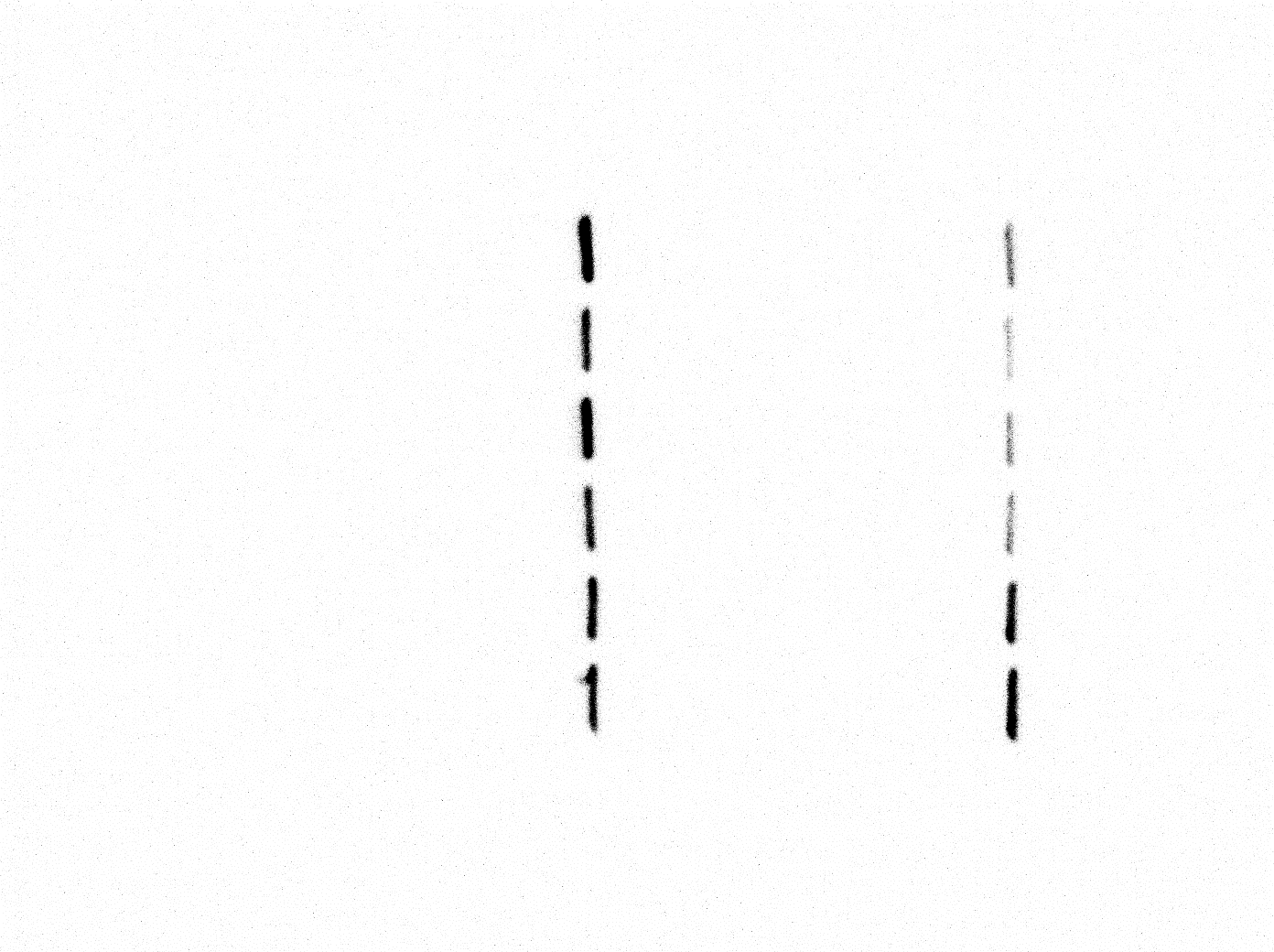


**Prdx4**


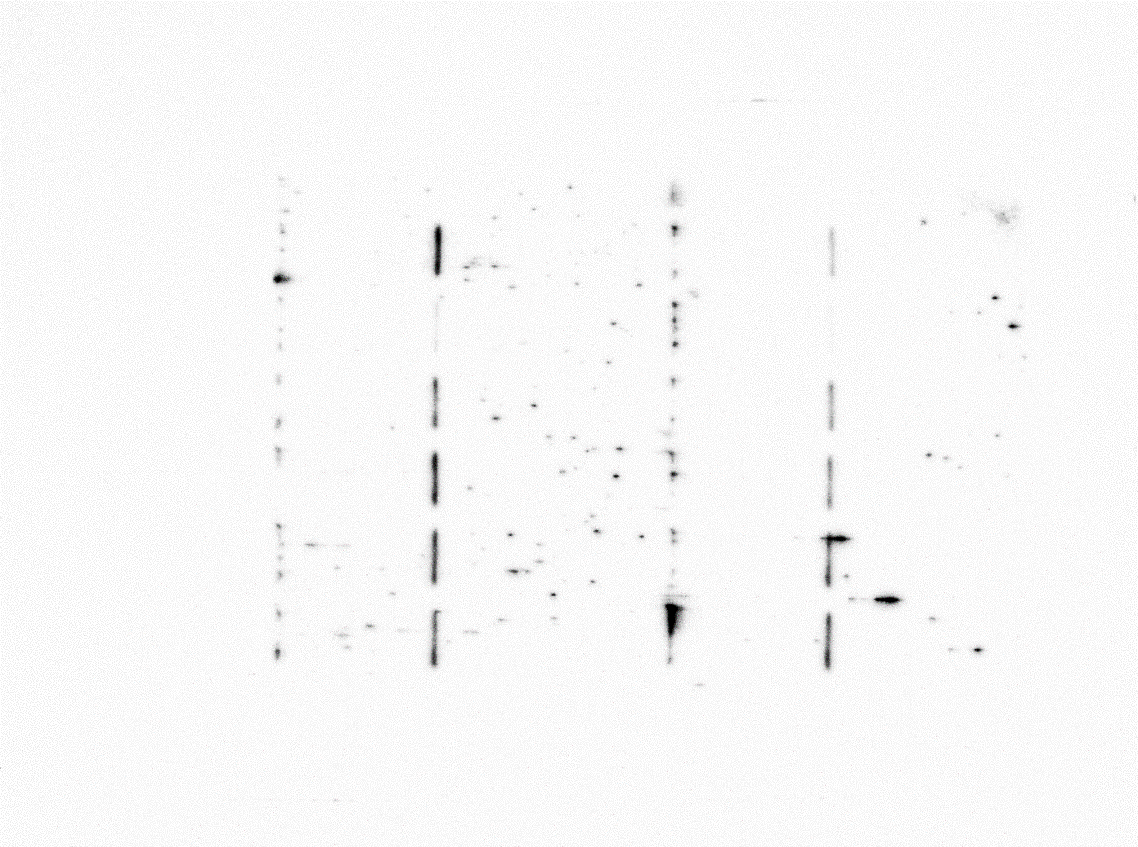

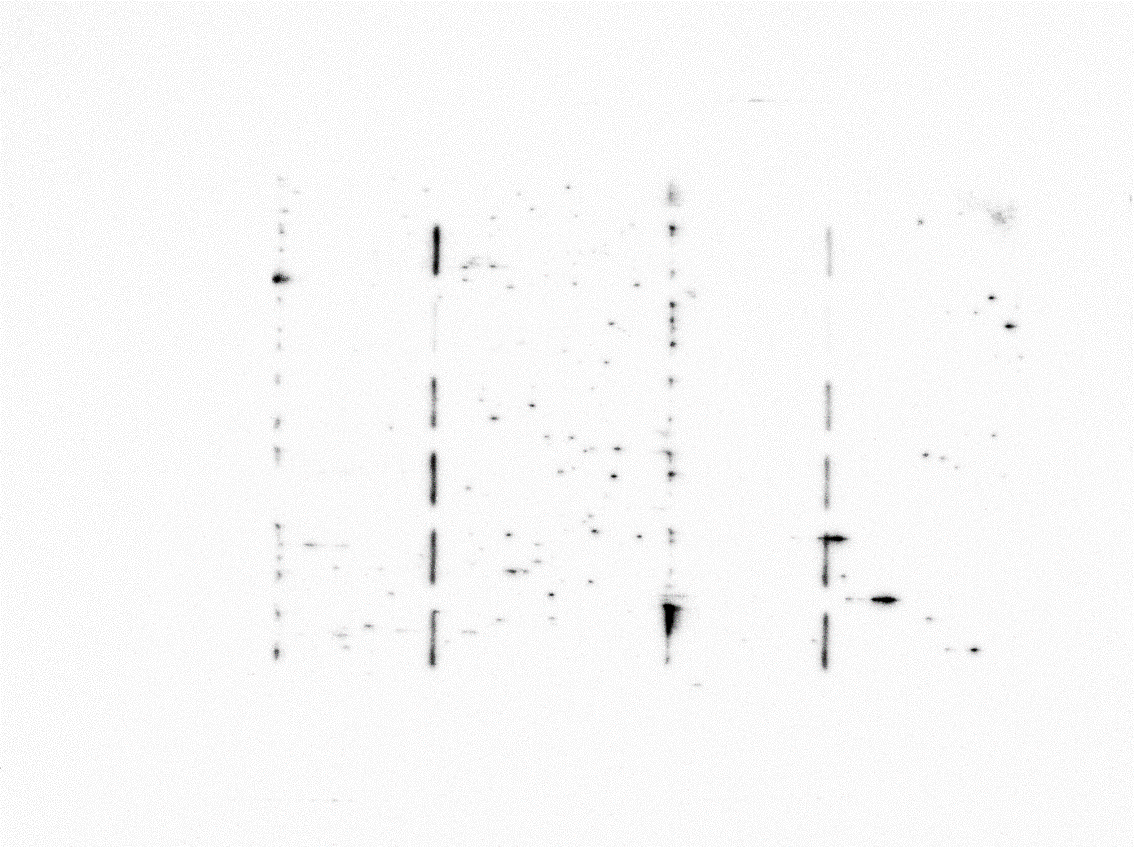


**Prdx5**


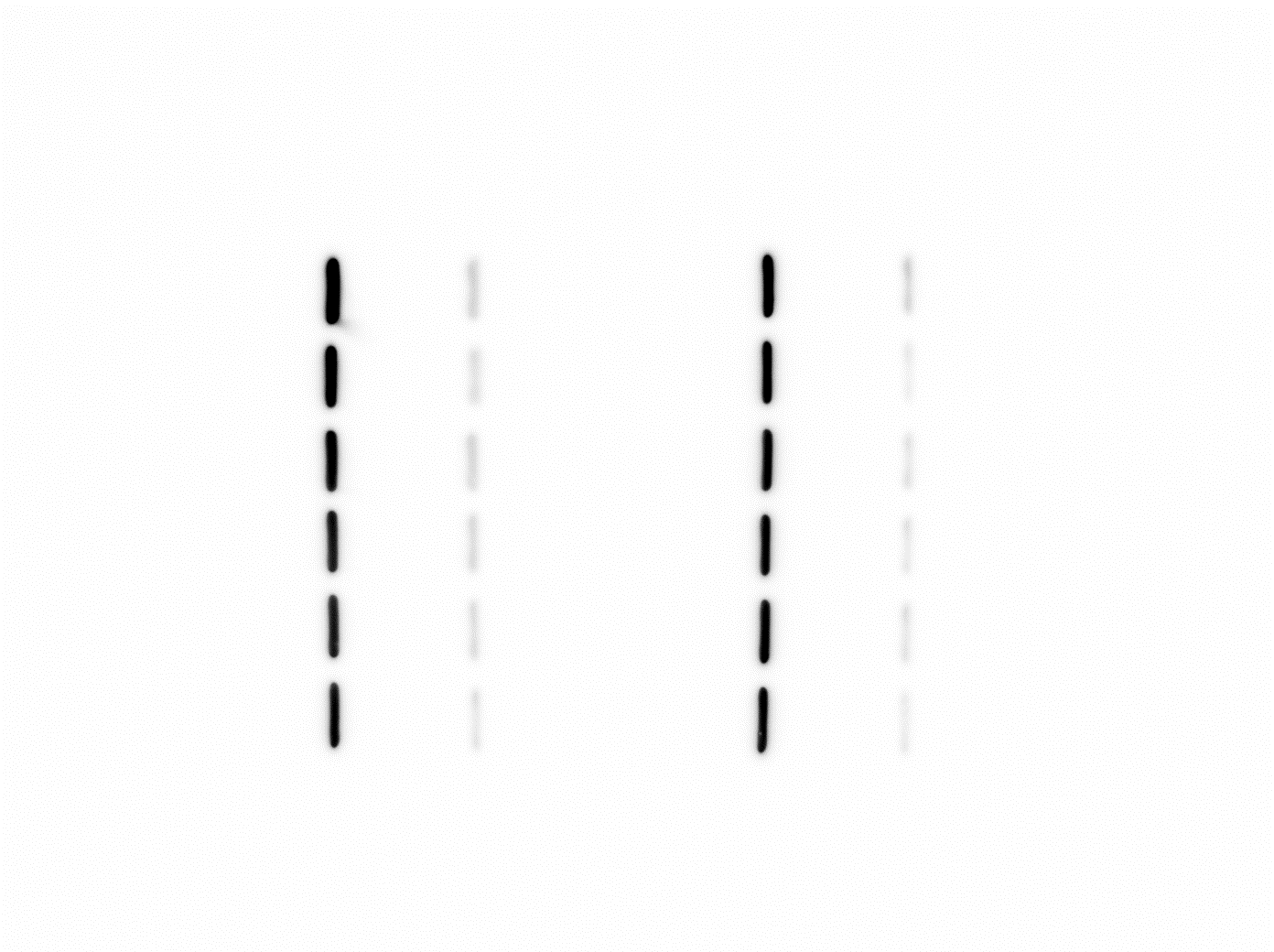

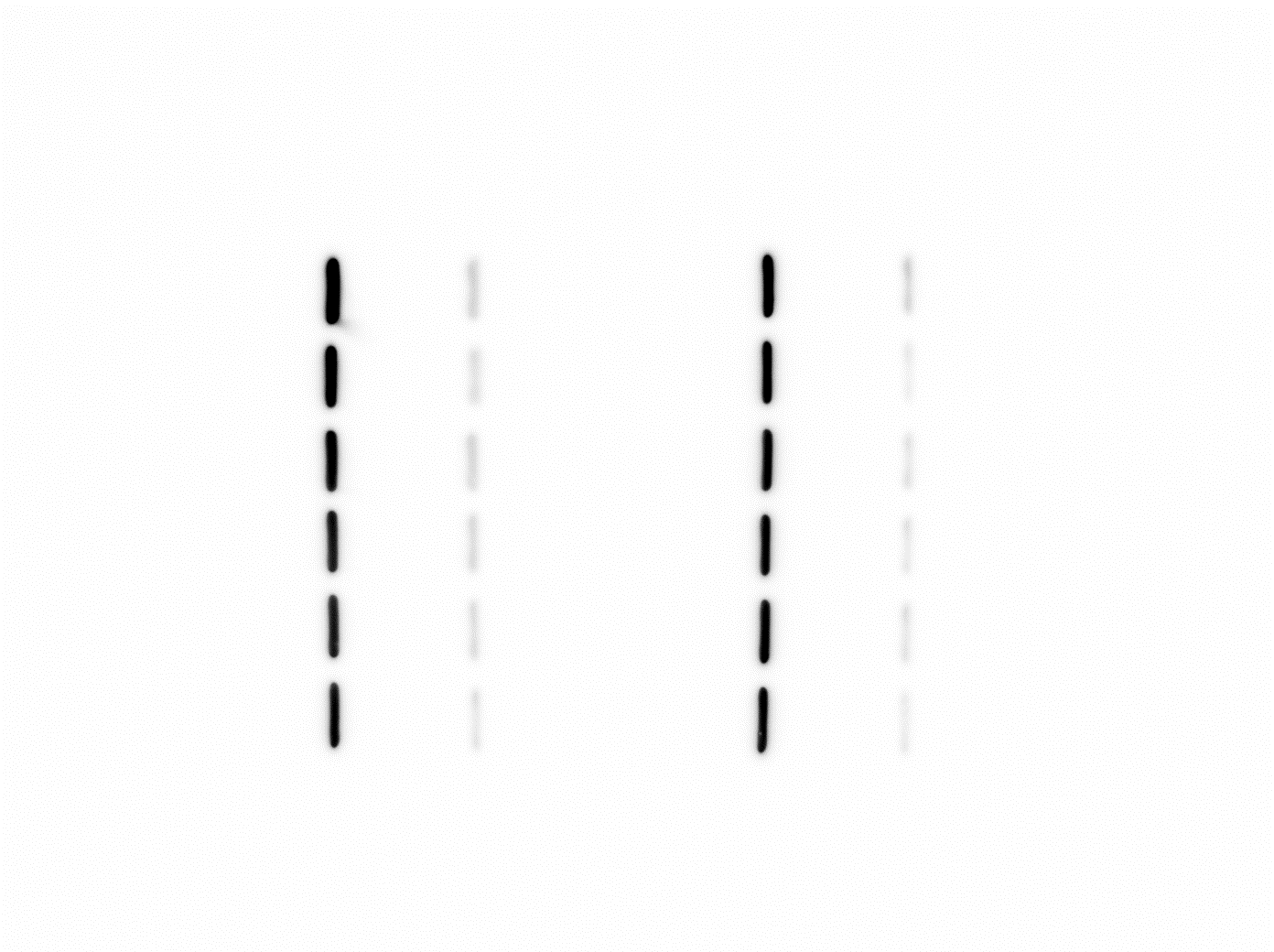


**β-actin**

**Prdx6**

**Figure 1– source data. Prdx 5 expression is controlled during bone cell differentiation.** (B) Protein levels of Prdxs in osteoblasts were determined via western blotting. (D) Protein levels of Prdxs in osteoclasts were determined using western blotting.
