# Supplementary figures and images for: Peroxiredoxin 5 regulates osteogenic differentiation through interaction with hnRNPK during bone regeneration
Source: eLife. 2023 Feb 3;12:e80122. doi: 10.7554/eLife.80122 (PMC9897727; doi:10.7554/eLife.80122)

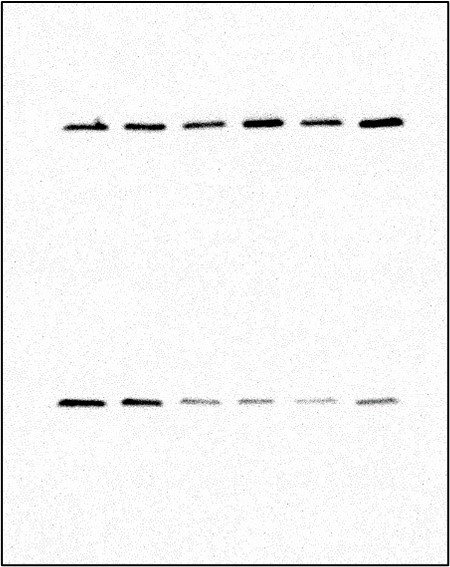

Supplement: Figure 1—source data 2. [file elife-80122-fig1-data2.zip › Figure 1-source data 12.jpg]

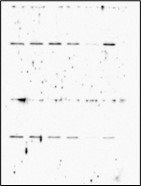

Supplement: Figure 1—source data 2. [file elife-80122-fig1-data2.zip › Figure 1-source data 13.jpg]

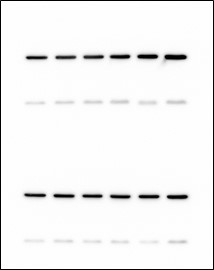

Supplement: Figure 1—source data 2. [file elife-80122-fig1-data2.zip › Figure 1-source data 14.jpg]

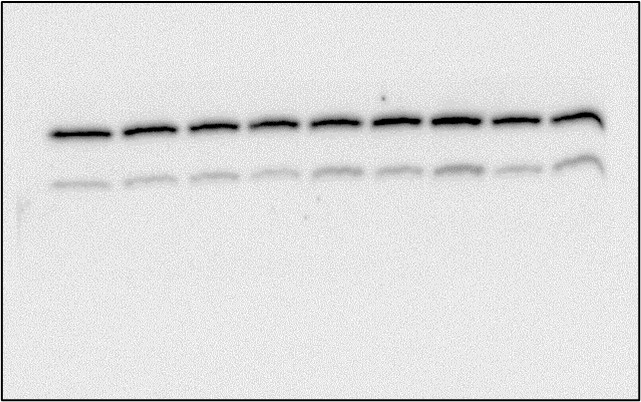

Supplement: Figure 1—source data 2. [file elife-80122-fig1-data2.zip › Figure 1-source data 1.jpg]

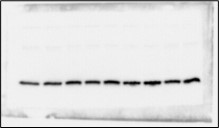

Supplement: Figure 1—source data 2. [file elife-80122-fig1-data2.zip › Figure 1-source data 2.jpg]

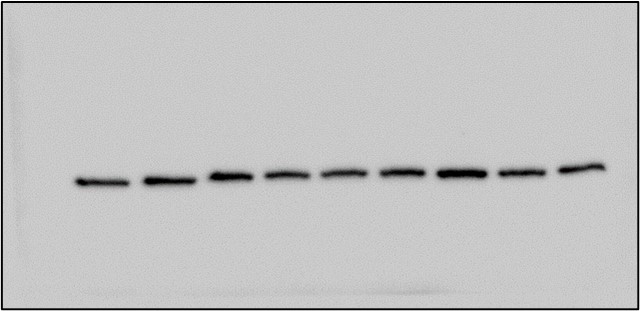

Supplement: Figure 1—source data 2. [file elife-80122-fig1-data2.zip › Figure 1-source data 3.jpg]

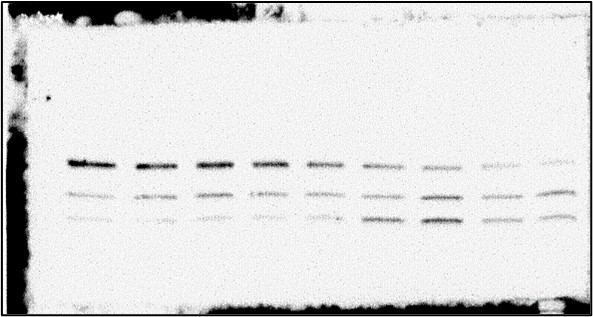

Supplement: Figure 1—source data 2. [file elife-80122-fig1-data2.zip › Figure 1-source data 4.jpg]

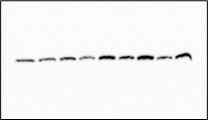

Supplement: Figure 1—source data 2. [file elife-80122-fig1-data2.zip › Figure 1-source data 5.jpg]

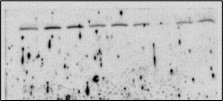

Supplement: Figure 1—source data 2. [file elife-80122-fig1-data2.zip › Figure 1-source data 6.jpg]

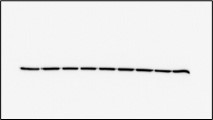

Supplement: Figure 1—source data 2. [file elife-80122-fig1-data2.zip › Figure 1-source data 7.jpg]

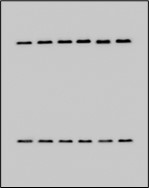

Supplement: Figure 1—source data 2. [file elife-80122-fig1-data2.zip › Figure 1-source data 8.jpg]

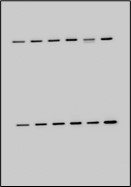

Supplement: Figure 1—source data 2. [file elife-80122-fig1-data2.zip › Figure 1-source data 9.jpg]

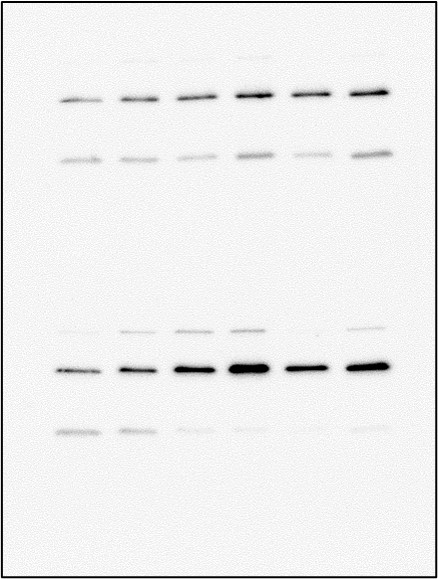

Supplement: Figure 1—source data 2. [file elife-80122-fig1-data2.zip › Figure 1-source data 10.jpg]

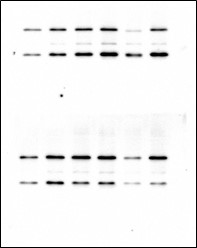

Supplement: Figure 1—source data 2. [file elife-80122-fig1-data2.zip › Figure 1-source data 11.jpg]

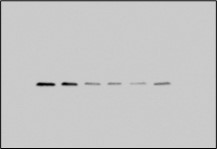

Supplement: Figure 2—source data 2. [file elife-80122-fig2-data2.zip › Figure 2-souce data 3.jpg]

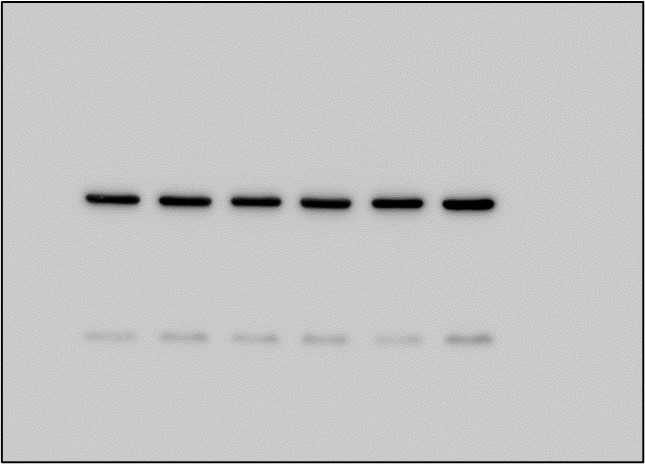

Supplement: Figure 2—source data 2. [file elife-80122-fig2-data2.zip › Figure 2-souce data 4.jpg]

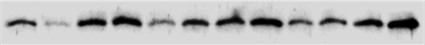

Supplement: Figure 2—source data 2. [file elife-80122-fig2-data2.zip › Figure 2-souce data 1.jpg]

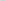

Supplement: Figure 2—source data 2. [file elife-80122-fig2-data2.zip › Figure 2-souce data 2.jpg]

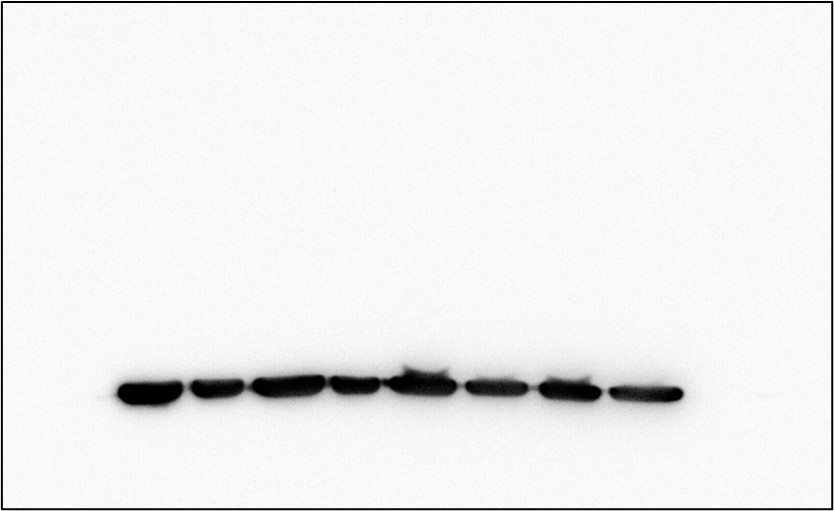

Supplement: Figure 3—figure supplement 2—source data 2. [file elife-80122-fig3-figsupp2-data2.zip › Figure 3-figure supplement 2-souce data 4.jpg]

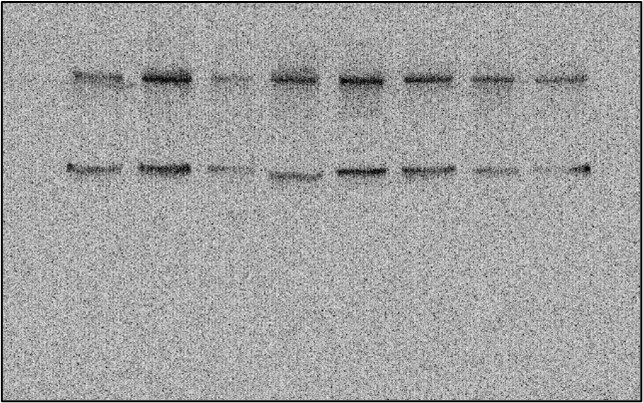

Supplement: Figure 3—figure supplement 2—source data 2. [file elife-80122-fig3-figsupp2-data2.zip › Figure 3-figure supplement 2-souce data 1.jpg]

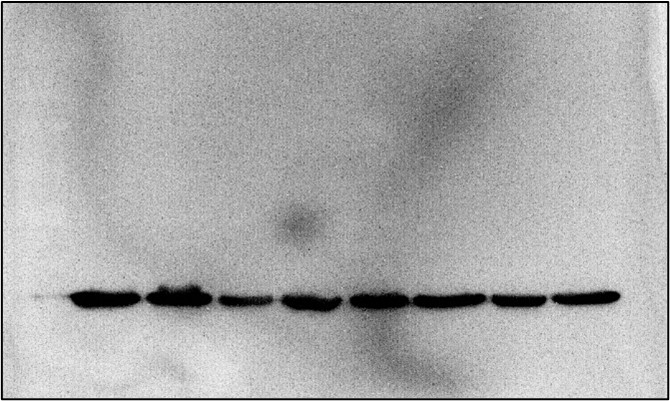

Supplement: Figure 3—figure supplement 2—source data 2. [file elife-80122-fig3-figsupp2-data2.zip › Figure 3-figure supplement 2-souce data 2.jpg]

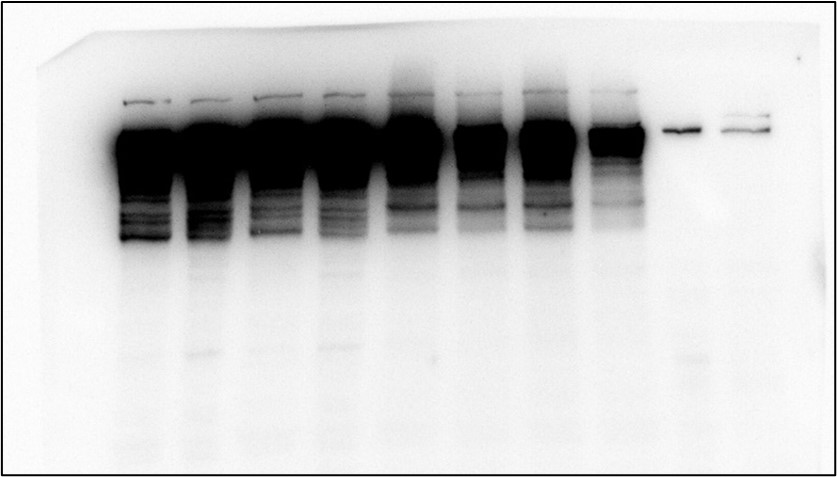

Supplement: Figure 3—figure supplement 2—source data 2. [file elife-80122-fig3-figsupp2-data2.zip › Figure 3-figure supplement 2-souce data 3.jpg]

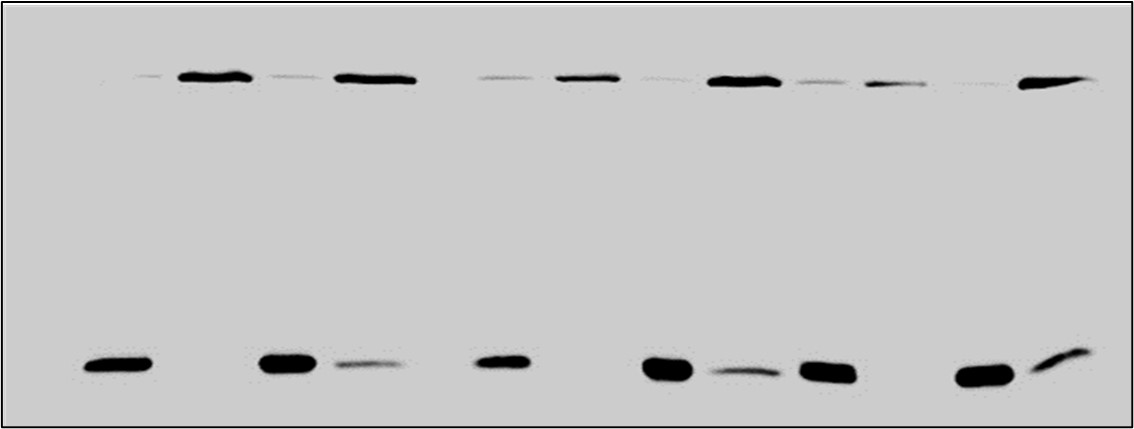

Supplement: Figure 5—figure supplement 1—source data 2. [file elife-80122-fig5-figsupp1-data2.zip › Figure 5-figure supplement 1-souce data 2.jpg]

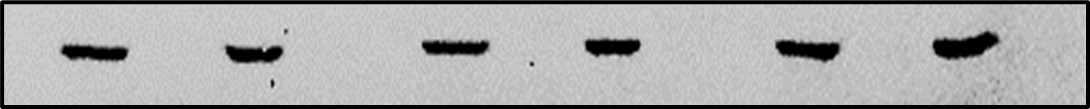

Supplement: Figure 5—figure supplement 1—source data 2. [file elife-80122-fig5-figsupp1-data2.zip › Figure 5-figure supplement 1-souce data 3.jpg]

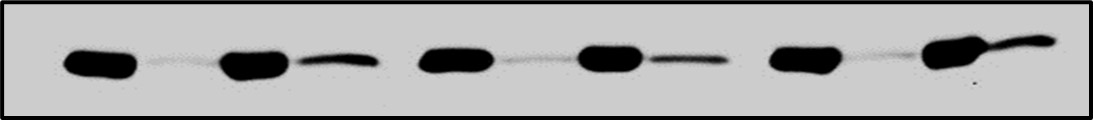

Supplement: Figure 5—figure supplement 1—source data 2. [file elife-80122-fig5-figsupp1-data2.zip › Figure 5-figure supplement 1-souce data 1.jpg]

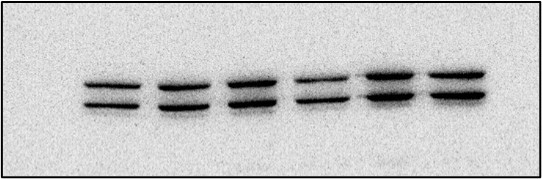

Supplement: Figure 6—source data 2. [file elife-80122-fig6-data2.zip › Figure 6-souce data 7.jpg]

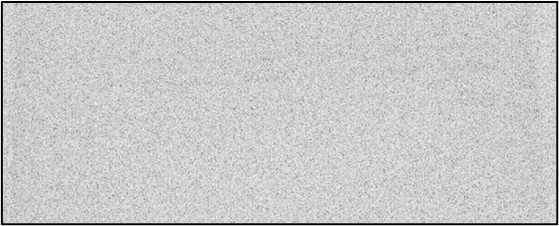

Supplement: Figure 6—source data 2. [file elife-80122-fig6-data2.zip › Figure 6-souce data 8.jpg]

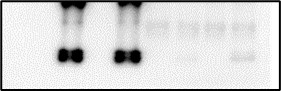

Supplement: Figure 6—source data 2. [file elife-80122-fig6-data2.zip › Figure 6-souce data 1.jpg]

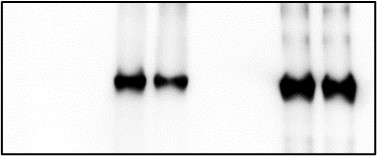

Supplement: Figure 6—source data 2. [file elife-80122-fig6-data2.zip › Figure 6-souce data 2.jpg]

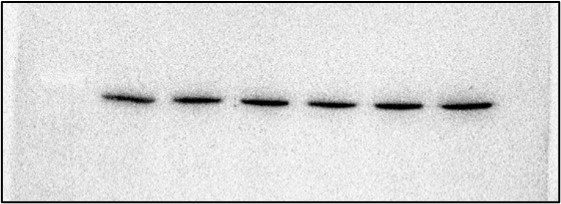

Supplement: Figure 6—source data 2. [file elife-80122-fig6-data2.zip › Figure 6-souce data 3.jpg]

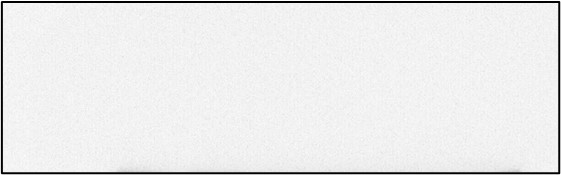

Supplement: Figure 6—source data 2. [file elife-80122-fig6-data2.zip › Figure 6-souce data 4.jpg]

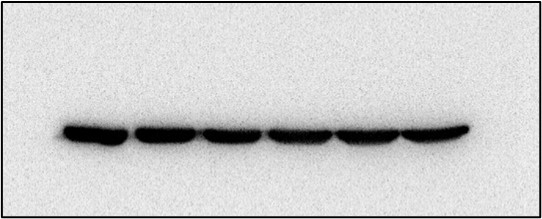

Supplement: Figure 6—source data 2. [file elife-80122-fig6-data2.zip › Figure 6-souce data 5.jpg]

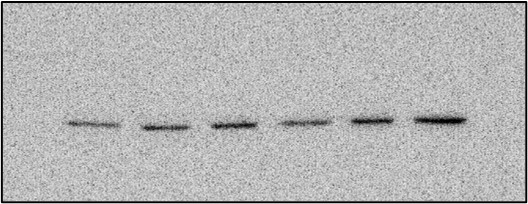

Supplement: Figure 6—source data 2. [file elife-80122-fig6-data2.zip › Figure 6-souce data 6.jpg]
