# Supplementary material for: Peroxiredoxin 5 regulates osteogenic differentiation through interaction with hnRNPK during bone regeneration
Source: eLife. 2023 Feb 3;12:e80122. doi: 10.7554/eLife.80122 (PMC9897727; doi:10.7554/eLife.80122)
Supplement: Figure 2—source data 1. [file elife-80122-fig2-data1.docx]

**Figure 2 – source data 1**

**A**

**BMP2**

**0 4 7 14 Days**


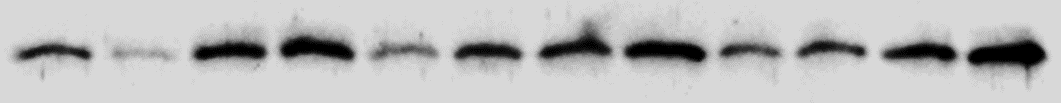

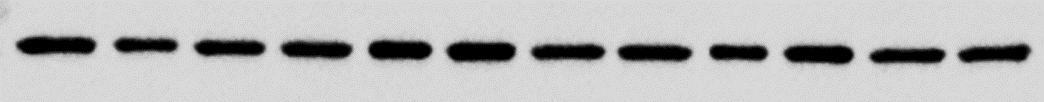


**D**

**RANKL 0 1 2 3 4 5 days**


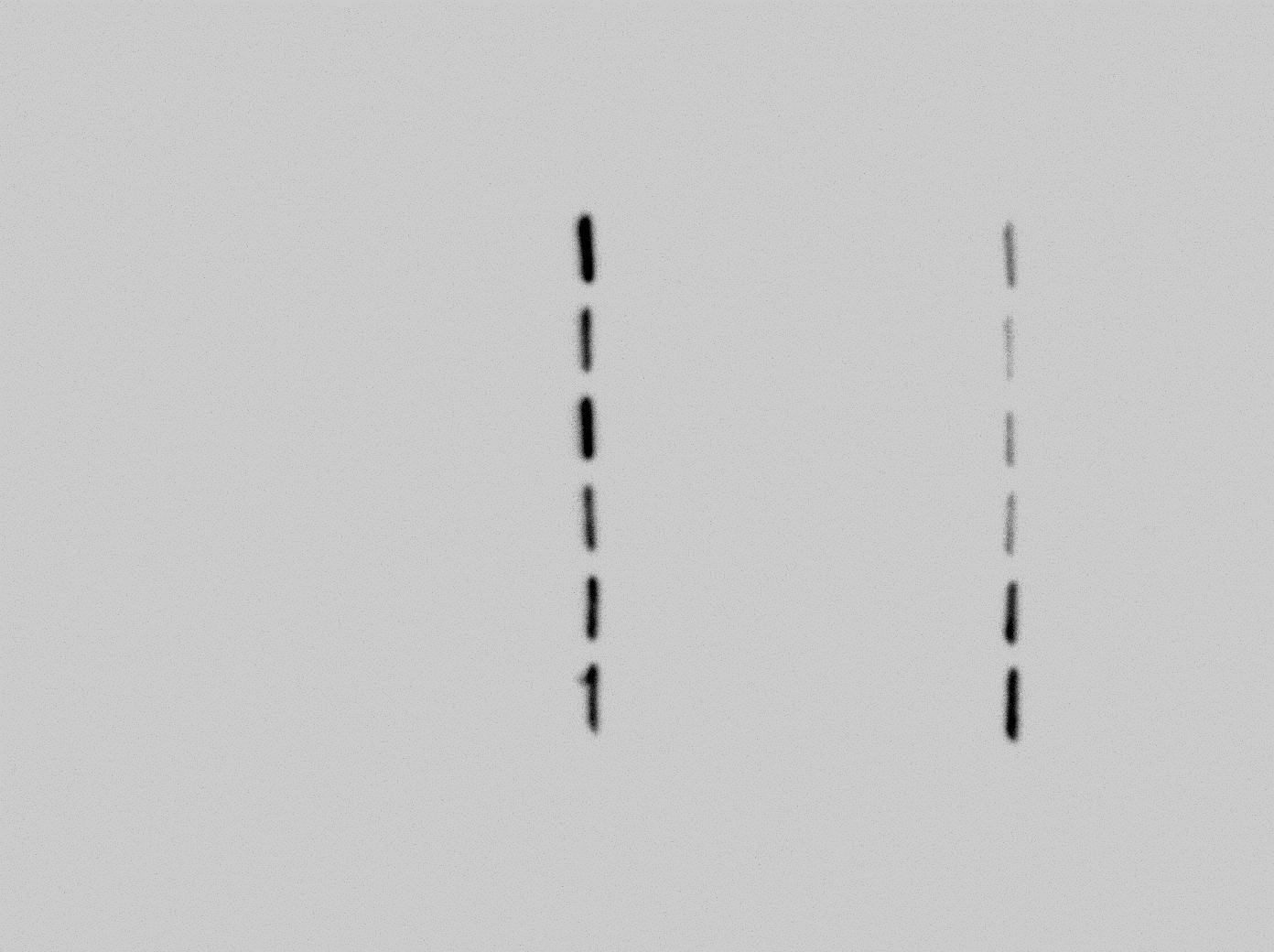


**Prdx5**


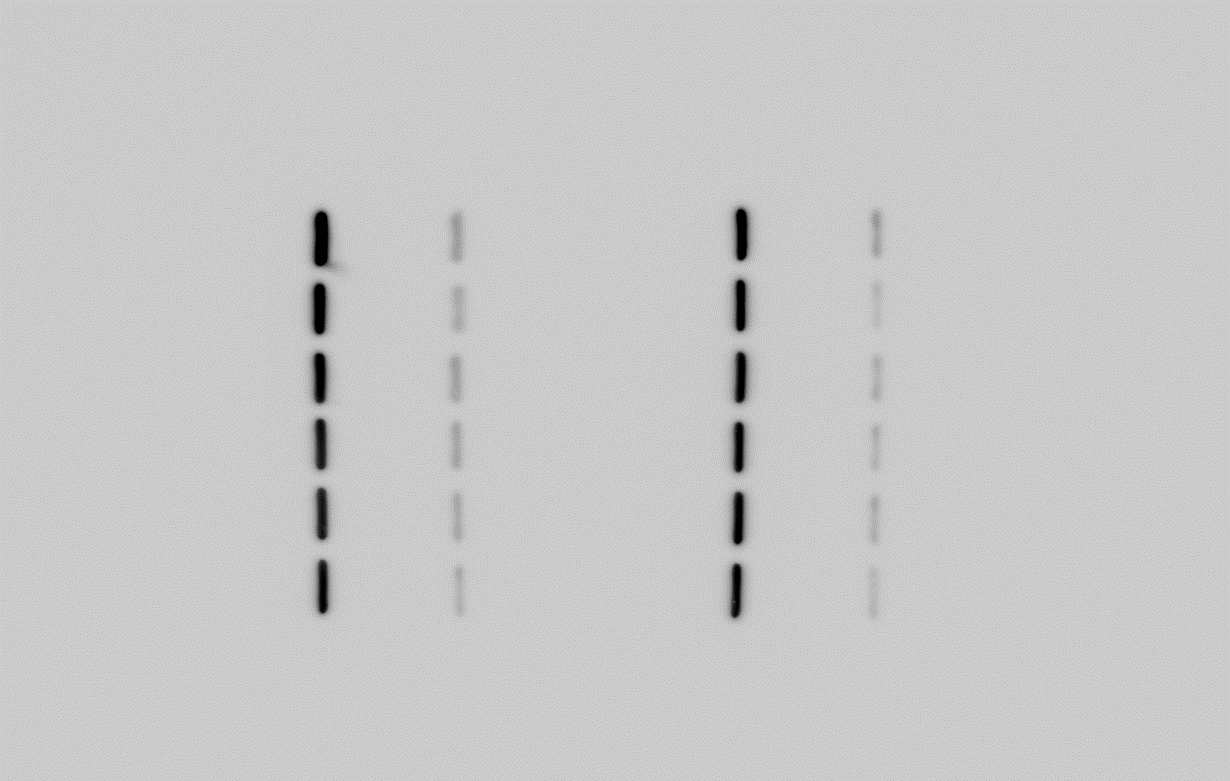


**β−actin**

**Figure 2 – source data.** (A) Western blotting was performed to determine Prdx5 expression during osteoblastogenesis. (D) Western blotting was performed to determine Prdx5 expression during osteoclastogenesis.
