# Supplementary material for: Peroxiredoxin 5 regulates osteogenic differentiation through interaction with hnRNPK during bone regeneration
Source: eLife. 2023 Feb 3;12:e80122. doi: 10.7554/eLife.80122 (PMC9897727; doi:10.7554/eLife.80122)
Supplement: Figure 3—figure supplement 2—source data 1. [file elife-80122-fig3-figsupp2-data1.docx]

**Figure 3–figure supplement 2- source data 1**

**B**

***Prdx5^Ko^***

**WT**

BMP2 - + - +


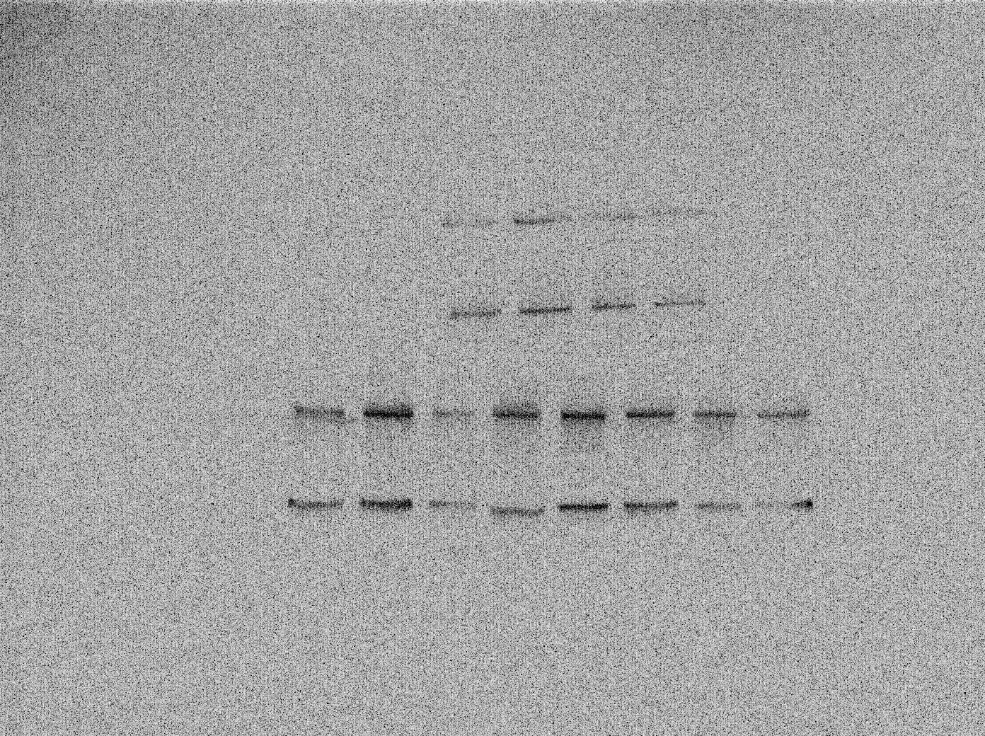


**AR**


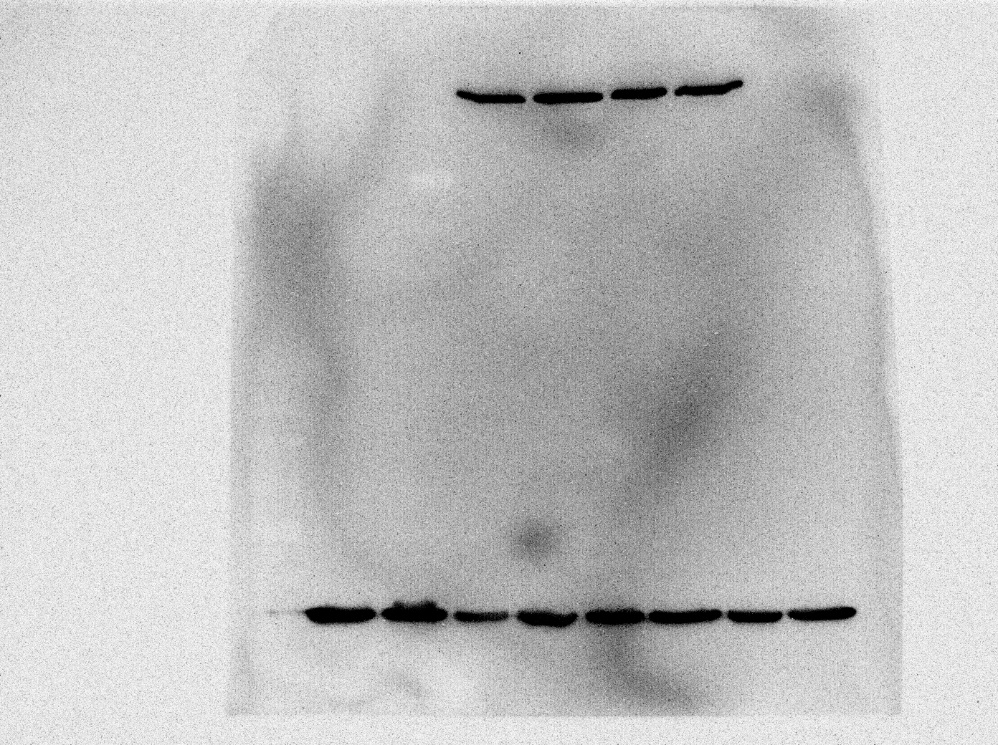


***Prdx5^Ko^***

**WT**

**β−actin**

RANKL - + - +

**C**


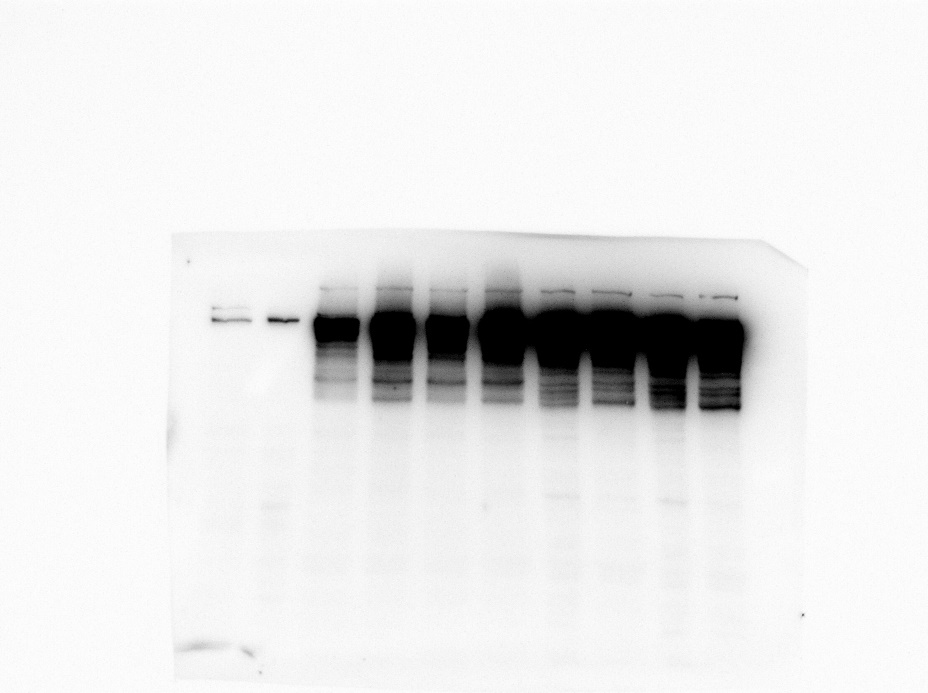

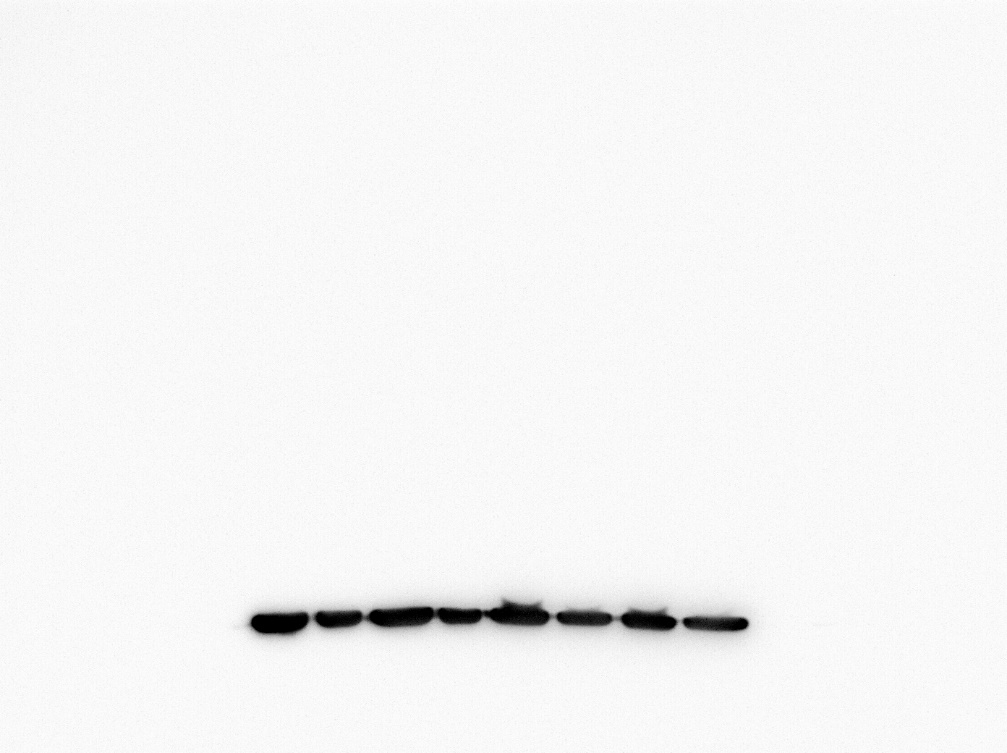


**AR**

**β−actin**

**Figure 3–figure supplement 2- source data. Testosterone and AR expression levels are suppressed in *Prdx5*^Ko^ male mice.** Protein levels (right figures) of androgen receptor (AR) were determined in osteoblasts stimulated with BMP2 for 7 days (B), and osteoclasts stimulated with RANKL for 3 days (C).
