# Supplementary material for: Peroxiredoxin 5 regulates osteogenic differentiation through interaction with hnRNPK during bone regeneration
Source: eLife. 2023 Feb 3;12:e80122. doi: 10.7554/eLife.80122 (PMC9897727; doi:10.7554/eLife.80122)
Supplement: Figure 5—figure supplement 1—source data 1. [file elife-80122-fig5-figsupp1-data1.zip › F5SD1.docx]

**Figure 5 – figure supplement 1 - source data**

**A**

BMP2

Nucl.

Cyto.

Nucl.

Cyto.

PBS


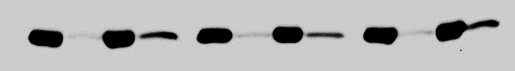


**Prdx5**


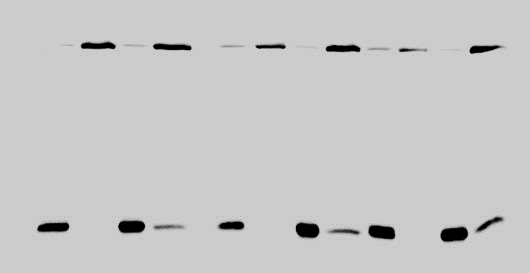


**lamin B**


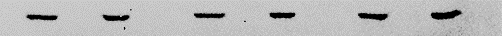


**α-tubulin**

**Figure 5 – figure supplement 1 - source data. BMP2 induces nuclear translocation of Prdx5.** (A) Western blot analysis of Prdx5 in the cytoplasmic and nuclear fractions of osteoblasts treated with BMP2 for 4days.
