# Supplementary material for: Peroxiredoxin 5 regulates osteogenic differentiation through interaction with hnRNPK during bone regeneration
Source: eLife. 2023 Feb 3;12:e80122. doi: 10.7554/eLife.80122 (PMC9897727; doi:10.7554/eLife.80122)
Supplement: Figure 6—source data 1. [file elife-80122-fig6-data1.docx]

**Figure 6 – source data**

**B**

Input IP-Flag

HA-Prdx5 - + - + - + - +


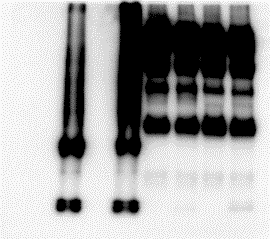
Flag-hnRNP K - - + + - - + +

HA


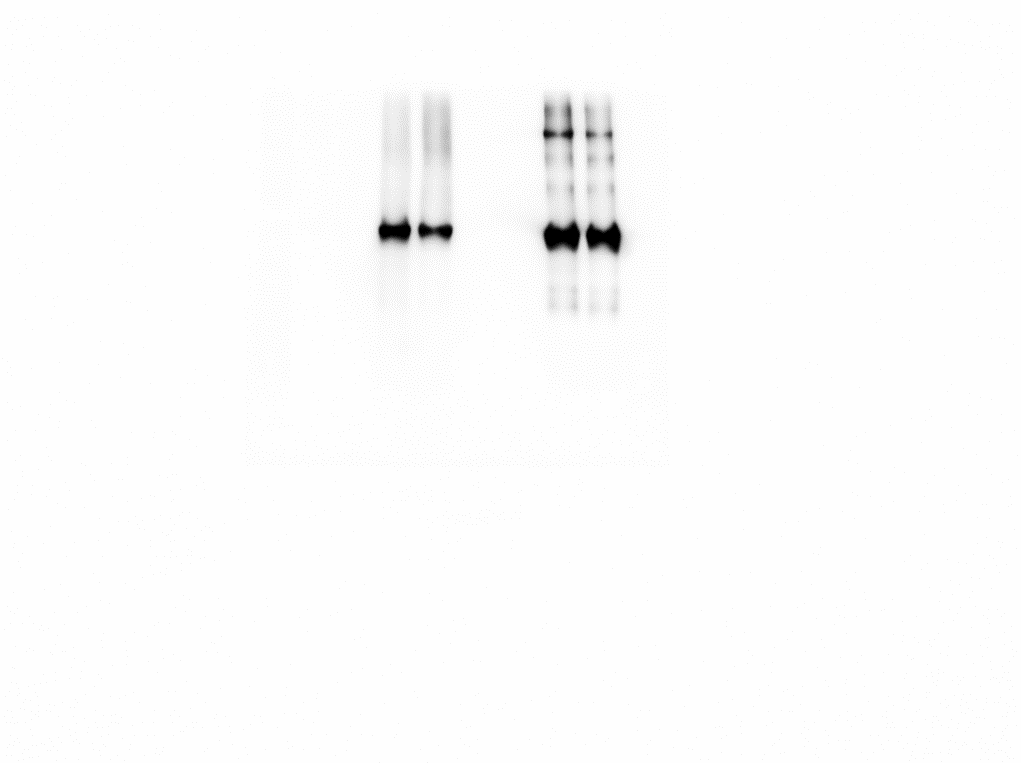


Flag

**D**

Cytoplasmic Nuclear

WT Ko WT Ko WT Ko WT Ko

- BMP2 - BMP2


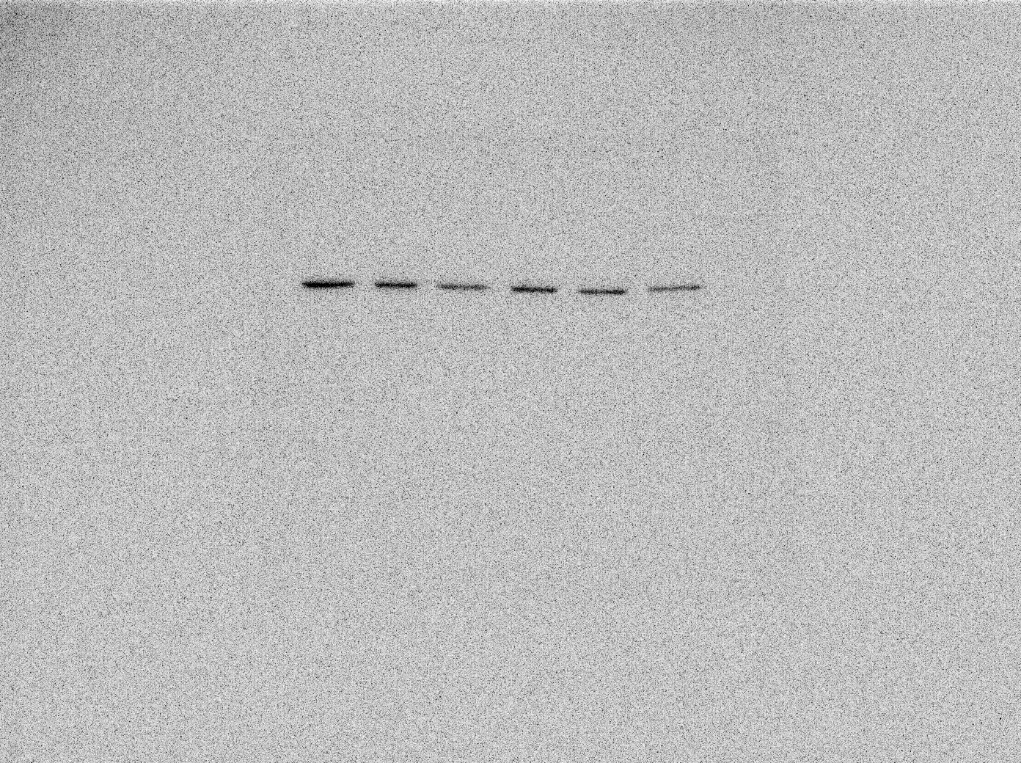

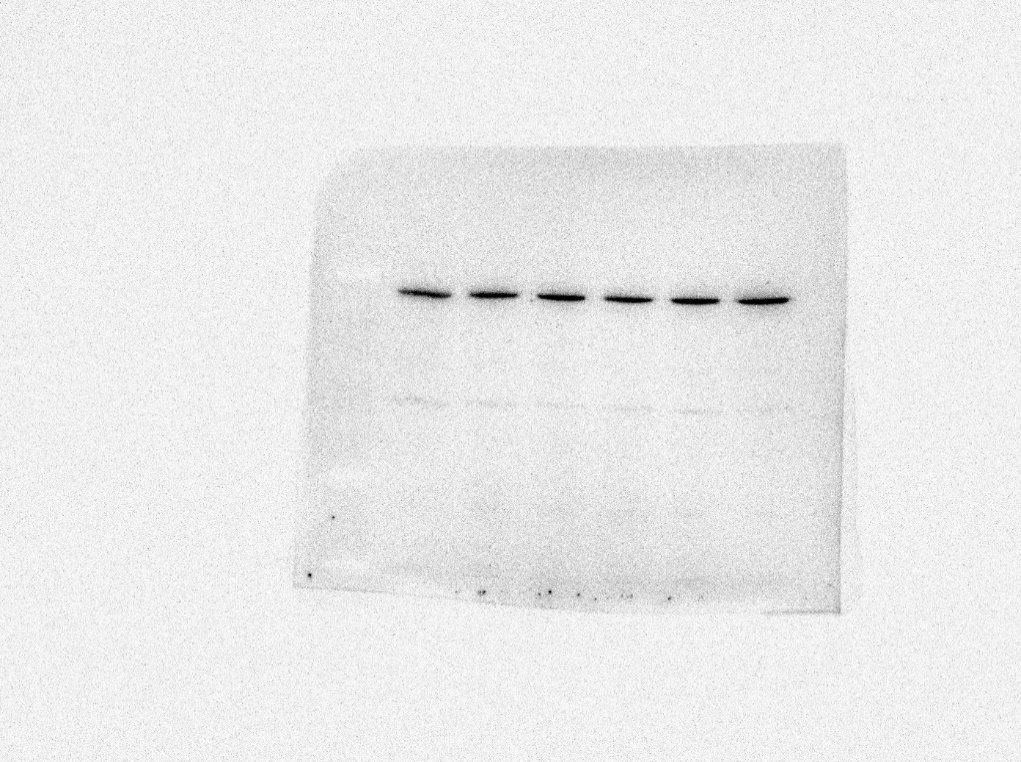


hnRNPK


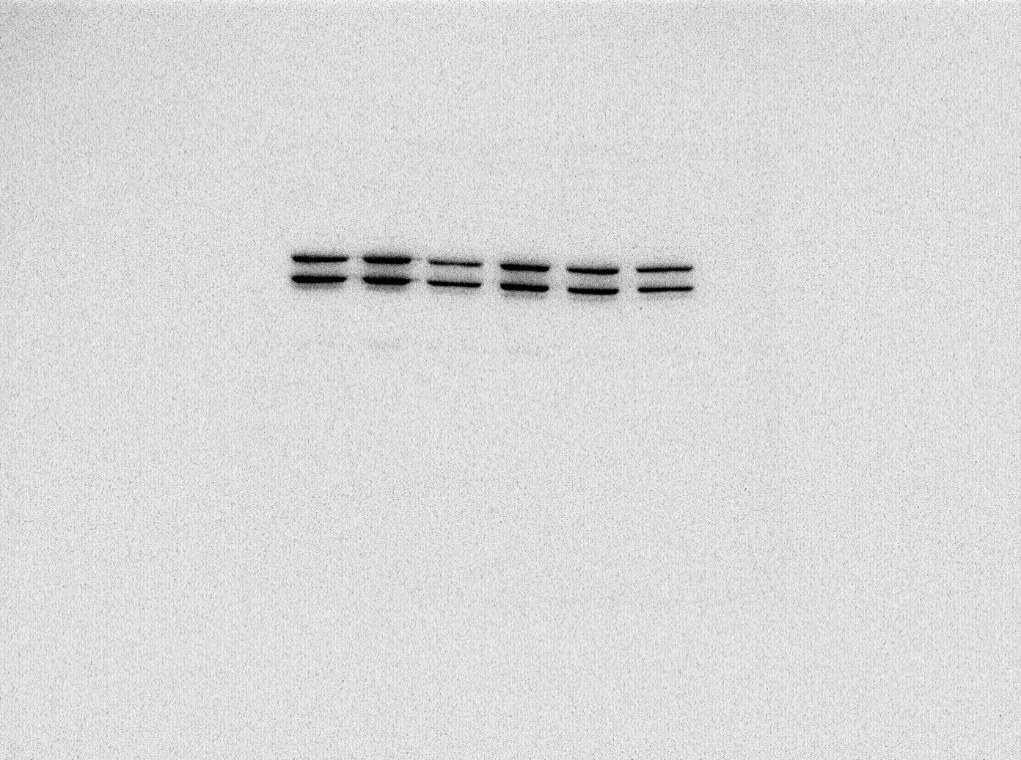

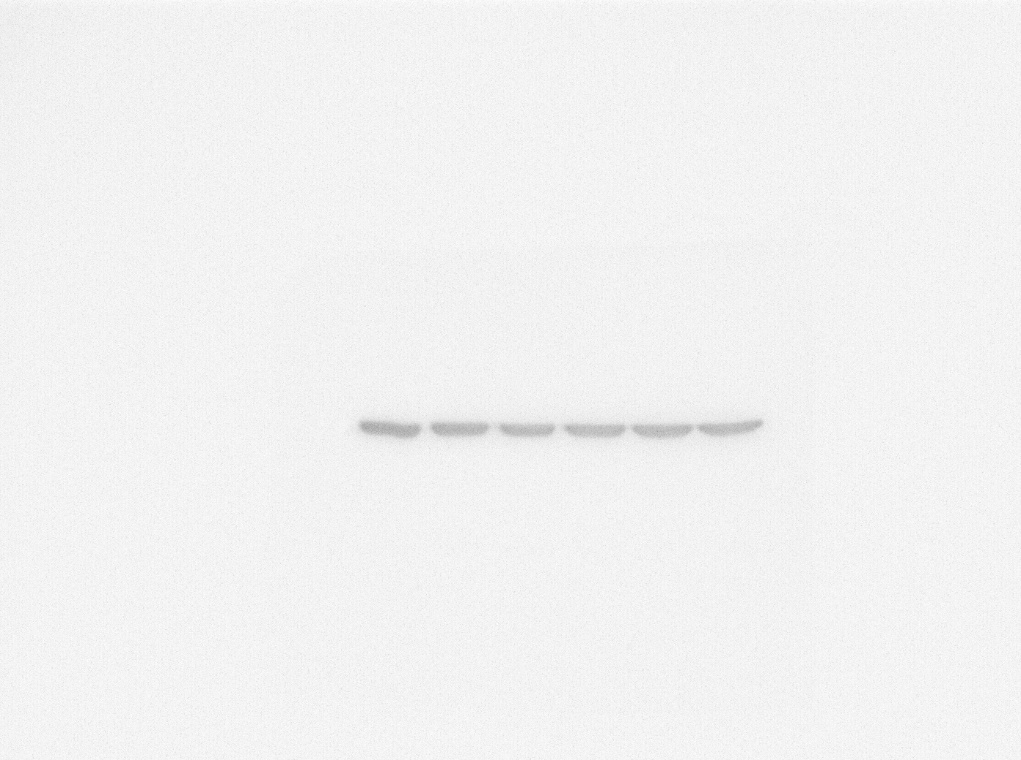


Lamin A/C


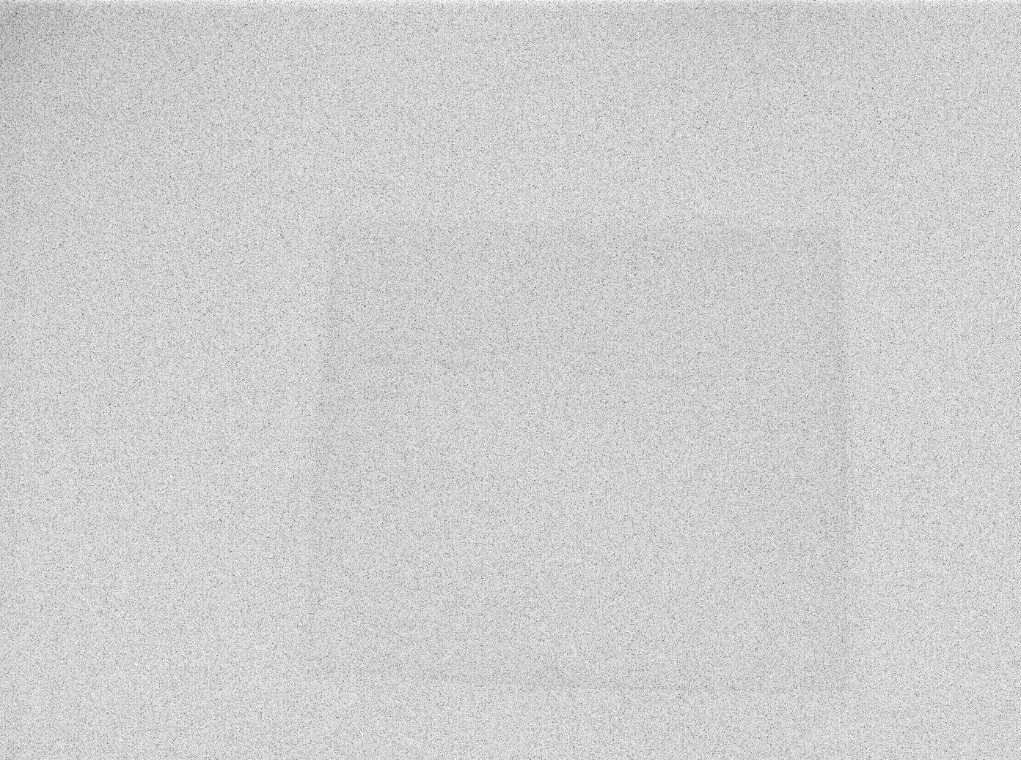

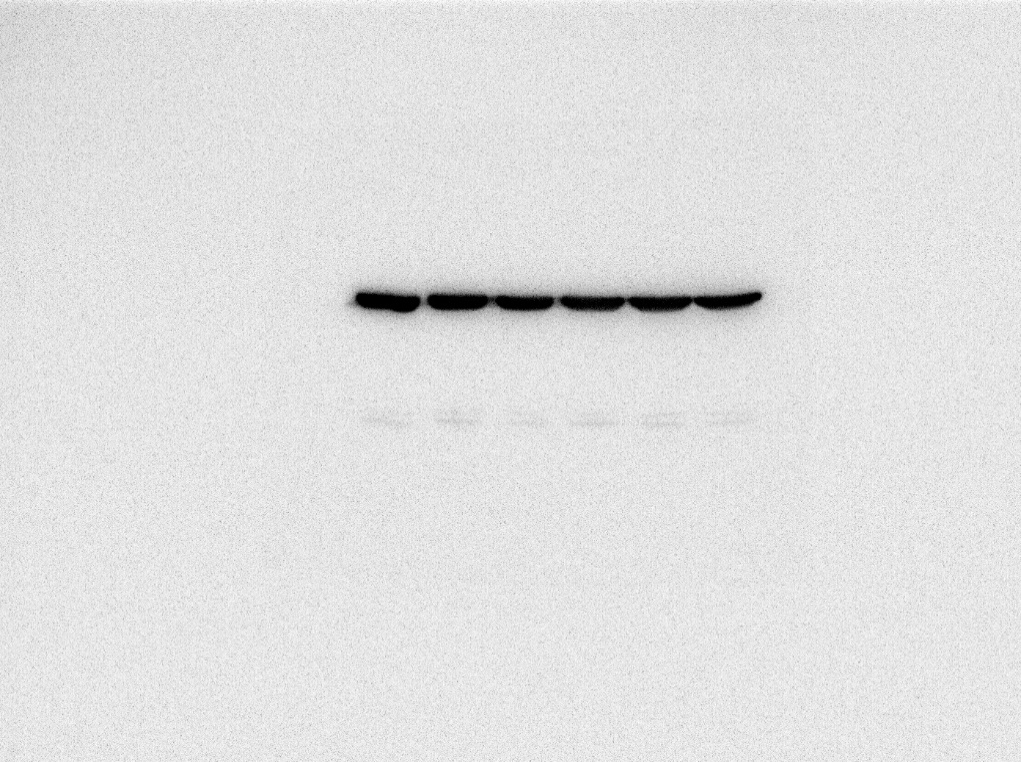


α-tubulin

**Figure 6 – source data. hnRNPK interacts with Prdx5 in osteoblasts.** (B) Immunoprecipitation (IP) was performed using HEK293T cells expressing various combinations of HA-tagged Prdx5 and flag-tagged hnRNPK. (D) hnRNAPK levels were determined in the cytoplasmic and nuclear fractions of WT, *Prdx5*^Ko^ cells. Osteoblasts were harvested on day 7.
